# Impact of Microwaves on Organic Synthesis and Strategies toward Flow Processes and Scaling Up

**DOI:** 10.1021/acs.joc.1c00865

**Published:** 2021-06-14

**Authors:** Katia Martina, Giancarlo Cravotto, Rajender S. Varma

**Affiliations:** † Dipartimento di Scienza e Tecnologia del Farmaco and Centre for Nanostructured Interfaces and Surfaces (NIS), University of Turin, University of Turin, via P. Giuria 9, 10125 Turin, Italy; ‡ Regional Centre of Advanced Technologies and Materials, Palacký University in Olomouc, Šlechtitelů 27, 783 71 Olomouc, Czech Republic

## Abstract

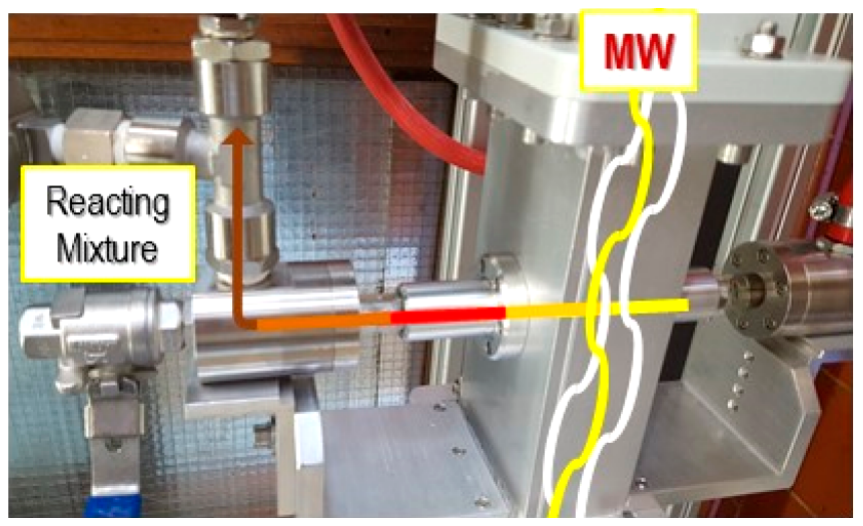

Microwave-assisted organic synthesis has been widely studied and deliberated, opening up some controversial issues as well. Nowadays, microwave chemistry is a mature technology that has been well demonstrated in many cases with numerous advantages in terms of the reaction rate and yield. The strategies toward scaling up find an ally in continuous-flow reactor technology comparing dielectric and conductive heating.

In the more than 30 years of microwave (MW) chemistry, many green protocols in organic synthesis have been reported, showing significant advantages compared to the conventional heating. Selective, volumetric dielectric heating has resulted in time and energy savings, enabled the deployment of safer solvents or solvent-free processes, selective catalysis in fewer steps, and generally attaining higher selectivity and yields.^1^ Although in the beginning, an improper temperature measurement sometimes overestimated the positive effect on the reaction rate, a fast and efficient energy transfer was demonstrated; however, for years, MW applications in organic synthesis were limited to the laboratories of a limited number of experts. The paucity of data available pertaining to the dielectric properties of materials as a function of temperature and frequency generated an empirical approach. In the meantime, the design of MW reactors was mainly focused on applications in analytical chemistry because of the rapid diffusion in sample mineralization. The lack of dedicated equipment often led to scarcely reproducible results and varying kinetics, which limited scaling-up studies for the industrial applications.

Despite the evident production efficiency under dielectric heating, the interest in industrial processing was delayed by the demand for safer equipment and plants.^2^ The candidates for further development were the most efficient and reproducible protocols.

However, continuous-flow synthetic processes were shown to be less flexible when working with different reactions. After the optimization of the residence time, mixing, and flow rate, the reaction could be easily scaled up with parallel reactors or in bigger size. In all cases, the main goal was to preserve the reaction kinetics.^3^

Aiming to improve heat and mass transfer, a progressive transition from batch to continuous processing is expected, which is a mandatory condition for exploiting the MW energy in industrial production. MW-assisted continuous-flow organic synthesis (MACOS) has been successfully investigated in a plethora of organic reactions (e.g., heterocycle synthesis and metal-catalyzed chemistry) and, in particular, for the semi-industrial preparation of aromatic compounds.^4^ Numerous studies on MW-assisted synthesis have nowadays revealed the possibility of transferring the methodology from the MW batch mode to a conventionally heated or MW-heated flow mode.

Because this field of research has been previously reviewed and a number of publications have reported the advances in MW-assisted organic synthesis (MAOS) in batch and in continuous flow mode,^5−10^ this Perspective has focused on recent studies with an emphasis on the advantages and limitations of MW heating. In this regard, synthetic protocols aimed at scaling up of the process and the comparison between different approaches directed to large-scale synthesis have been selected to provide a comprehensive picture of the state of the art. Recent advancements in stereoselective MAOS are included as well.

## Recent Advances in Microwave Technology

The use of alternative energy sources to improve mass or heat transfer has tremendous potential for chemical reactions, and dielectric heating may strongly enhance the reaction rate. Scrutinizing the chemical engineering advancements to intensify synthetic protocols under MW irradiation, special attention is being paid to the design of the MW cavities that provides a homogeneous field and uniform heating.^11^

Single-mode MW instruments have higher energy efficiency when compared to multimode counterparts, as the reactor vessel geometry and position enable a well-defined field. A monomodal MW instrument is well suited for the mechanism study of the chemical reaction, but only relatively small vial diameters may receive/adsorb significant power.^12^

**Figure 1:**
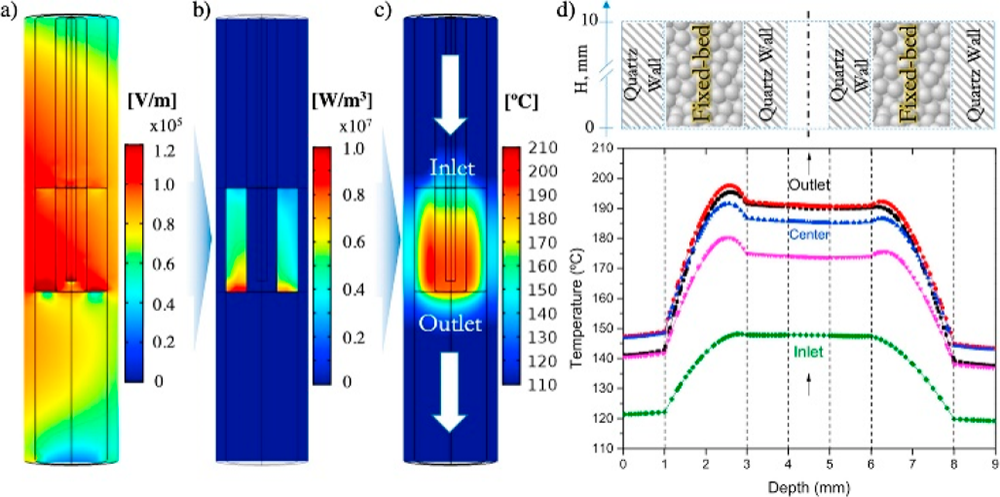
Simulated view of the quartz tube fixed bed of NaY zeolite and its corresponding (a) electric field, (b) power, and (c,d) spatial temperature distribution at steady state. Reproduced with permission from ref (13). Copyright 2019 Elsevier.

A recent study reported a fixed bed of 200 mg of NaY zeolite in a monomode rectangular resonant cavity wherein a temperature gradient was correlated to the nonuniform electric-field distribution.^13^ As presented in the Figure 1, the region at the higher temperature corresponds to the higher electric field region, and almost 60% of the sample has a temperature from 160 to 197 °C.

The attenuation of the electromagnetic wave, measured at a small penetration depth of the most adsorbing solvent, is another limitation of MW irradiation when large-scale synthesis is studied. Different approaches have been pursued to overcome this limitation, and the combination of MW irradiation with acoustic waves (ultrasound) may help generate an increase in the penetration depth beyond intensifying the mixing and mass transfer.^14,15^ The mechanical stirring would yield volumetric heating, and an example of a large-scale MW batch reactor devoted to synthesis has been described.^16^

Understanding how the MW deployment can enhance the reaction rates in organic synthesis requires a good background with knowledge of MW interactions with matter and other fundamentals. Moreover, a strict control of the applied energy with suitable hardware and software and reliable tools for temperature and pressure measurement is necessary. A rational balance of the pros and cons of MW energy deployment is relevant in deciding when and where MW irradiation can replace other energy input sources, and for this purpose, a study by Mase et al. reported a method that combines experimental design and 3D surface approximation for the rapid optimization of reaction conditions.^17^ The principles behind and the factors determining the successful scale-up of MW-assisted technology are the frequency, power, penetration depth, and energy consumption; thus studies in this field are collected in this comprehensive review focused on instrument design and reactor configuration.^18−20^

Modern MW technology can finely tune the electromagnetic radiation at high field strength and frequency, leading to fast and safe heating. MW heating can, a priori, be independent of the macroscopic temperature; MW penetrability is dependent on the frequency. National and international institutions allow only a few frequencies for laboratory, industry, and medical (ISM) applications. The main frequency bands for commercially available equipment are 2.45 GHz (most common), 915 MHz (USA), and 896 MHz (U.K.). Although equipment at 5.8 GHz is presently being created, an industrial setup requires further developmental work. MW irradiation at 5.8 GHz displays a shorter penetration depth but a better energy conversion. The selected frequency impacts the dielectric constants and the dissipation factors (tan δ). Apart from MW reactors working at 2.45 GHz, all other devices require ad hoc designs because they are not available from commercial suppliers.^21^

Relevant advances in the “kilolab” continuous-flow MW mode have been reported by Morschhäuser et al. using a transmission-line short-circuited waveguide unit, combining the features of mono- and multimode systems.^22,23^

**Figure 2:**
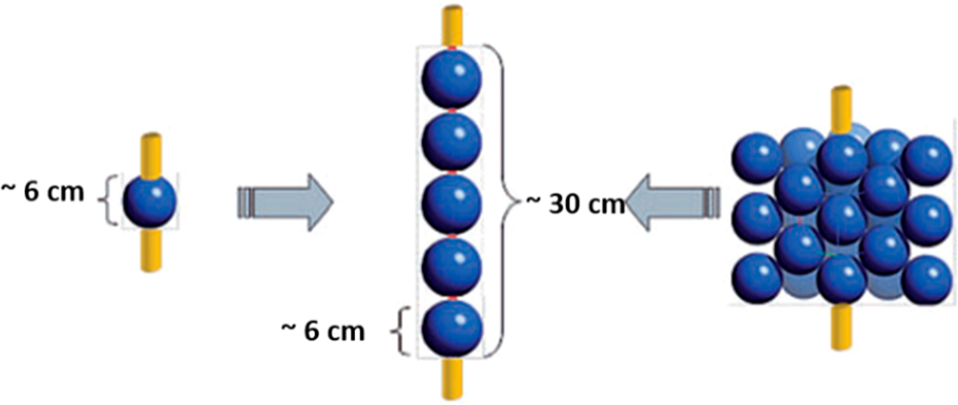
MW resonator flow concept obtained by combining mono- and multimode technologies. Reproduced with permission from ref (22). Copyright 2012 Walter de Gruyter.

The new generation of MW reactors have come a long way in terms of efficiency and compatibility with the continuous-flow systems; they can promote chemical reactions at varying temperatures up to 300 °C and pressures up to 200 bar with safety as a priority and excellent parameter controls.^24^ The combination of several modes in the direction of the reaction flow allows a homogeneous field distribution in resonator cavities, therefore, the continuous reactions have advantages related to MW irradiation and flow chemistry (Figure 2).

**Figure 3:**
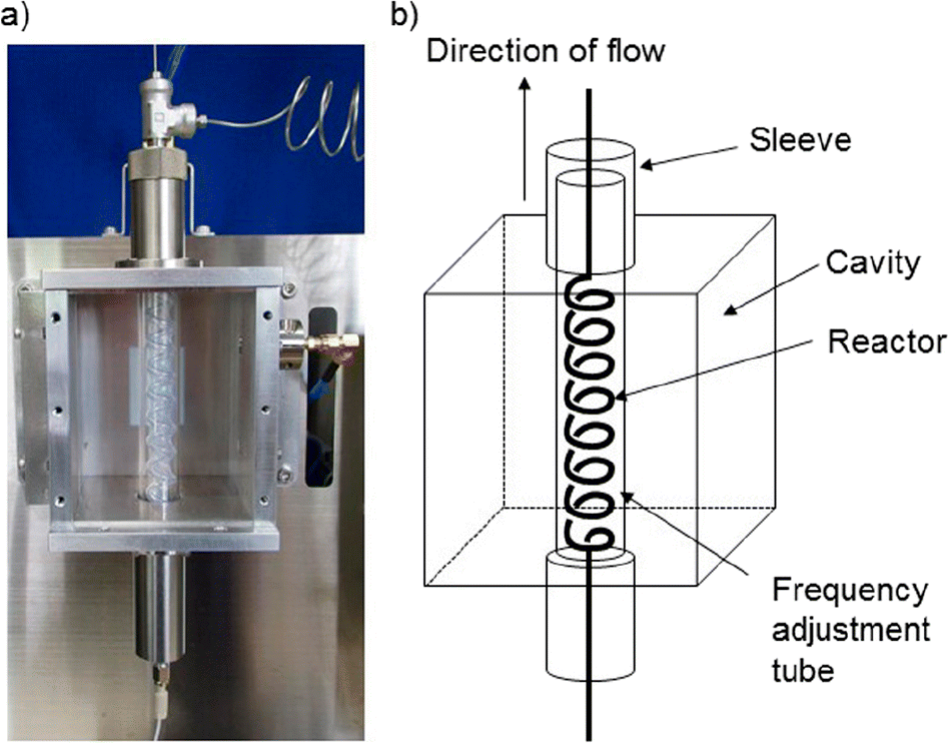
Photograph and schematic representation of the resonant-type MW tubular reactor. Reproduced with permission from ref (25). Copyright 2018 The Authors.

**Figure 4:**
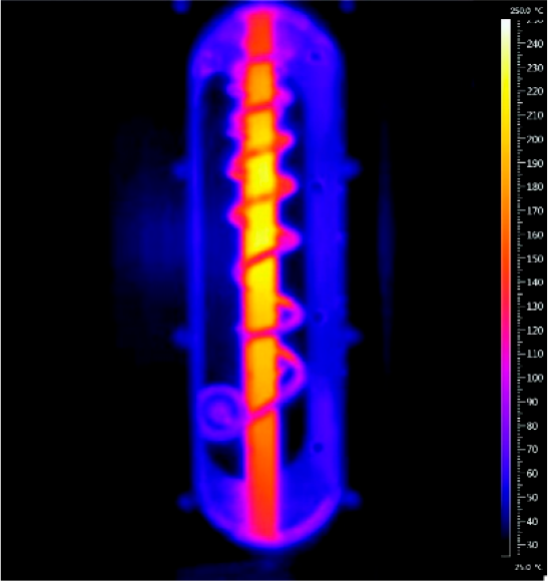
IR camera image of a continuous oxidative Heck reaction in DMF (200 °C set temperature). The reactor is heated by the helical antenna. Reproduced with permission from ref (26). Copyright 2012 American Chemical Society.

Two examples of the evaluation of MW applicators for flow synthesis are represented in Figures 3 and 4. A resonator-type MW reactor for continuous flow mode is depicted in Figure 3. The system comprises a rectangular MW cavity equipped with a helical tubular borosilicate glass reactor with an internal volume of 1.0 mL.^25^ In Figure 4, another example of an MW tubular reactor heated by means of an Al–Cu helical antenna is represented and, in particular, the IR camera image that represents the temperature is shown.^26^ The temperature is rather homogeneous, and lower temperatures are registered at the top and bottom of the reactor, which correspond to the entrance and exit of the flow.

Large laboratory MW units have been produced to achieve the kilogram scale in loop, in stopped flow or continuous flow, and with parallel vessels; nevertheless, these systems have a few inherent limitations.^27^

The industry is adopting strict strategies to minimize risks and to avoid batch failures, thus moving toward the continuous-flow synthetic processes. Proper modeling and simulation tools are fundamental for the design of safe and efficient reactors. This would help to get rid of bottlenecks that currently prevent scaling up. In addition to the reaction behavior during the reaction course, modern MW reactors should strictly monitor all of the parameters. Several variables affect the output of MW reactor and its scale, reflecting that MW technology cannot be implemented in a straightforward manner like classic conductive heating.

Industrial MW installations have been developed by specialized companies on the basis of the chemical conversion and the productivity required. Equipment manufacturers, who offer on-demand specifications (reactor geometry, frequency, power, etc.), can then make recommendations and offers.

## Impact of High Temperature and Pressure on Homogeneous Organic Synthesis

Recent examples of batch and continuous MW organic synthesis take advantage of modern instruments in which the temperature and pressure are controlled. Esterification, aromatic nucleophilic substitution, and Claisen rearrangement, among others, have been optimized under high pressure and consequently at higher temperature.

An investigation of Fisher esterification evidenced the importance of heating the solvent above the atmospheric boiling point, and for this reason, the flow reactor under pressure has been successfully exploited.^28^ Comparing different studies also underlined the importance of the MW reactor design, and the depth of the MW irradiation has sometimes been measured. The typical limitations due to MW absorbance and its conversion have been largely centered on continuous operations with a thinner reaction cell, tube, or tubular coil.

**Scheme 1:**
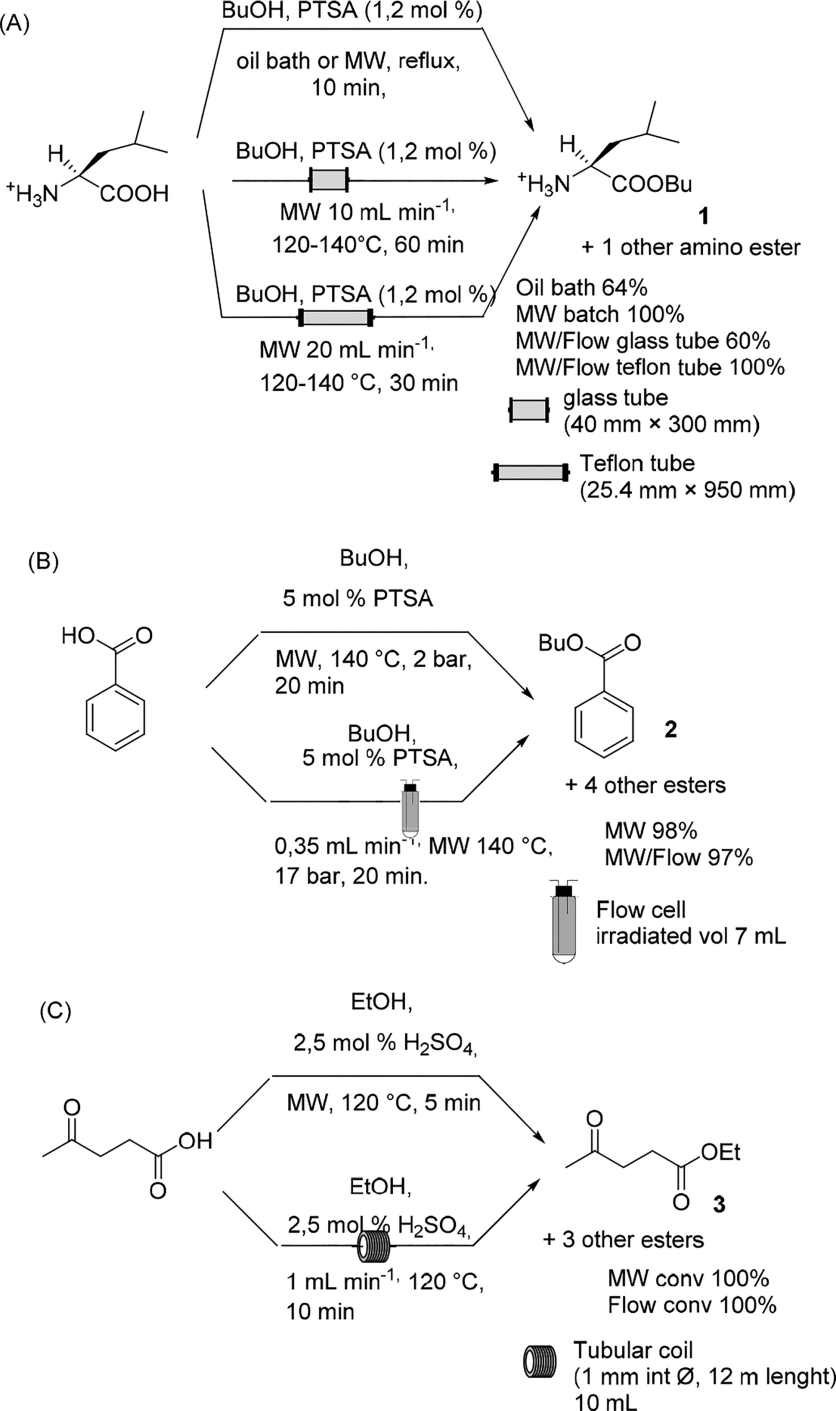
Fischer Esterification Reaction

In 2017, the esterification of leucine with PTSA (*p*-toluene sulfonic acid) and butanol was efficiently performed in an MW reactor (Scheme 1A, compound **1**).^29^ The authors compared the Fischer–Speier esterifications performed in a round-bottomed flask or in two types of tubular reactors of different inner diameters and lengths. By means of the in situ measurement of the dielectric properties of the reaction mixture in methanol, the penetration depth was calculated (∼13 mm), and the reaction was therefore performed in MW batch mode and continuous-flow mode with an MW oven equipped with tubular reactors made of glass or Teflon with varying dimensions (Scheme 1A). In the case of a large-scale reaction with a Teflon tube deployment, the authors observed that the highest impact on the reaction conversion was correlated to the inner diameter of the reactor. With a 40 mm internal diameter, the beneficial effect of flow was apparent, but the reaction was not complete. As depicted in Scheme 1A, the full conversion was attained when the MW batch reaction was performed at reflux with a thinner tubular reactor heated to 120–140 °C. Esterification that was performed in a conventional batch mode gave only 64% conversion. Furthermore, this study demonstrated that the MW-assisted flow reaction could be successfully scaled up to a tubular system of a ∼600 mL inner volume with a flow of 20 mL·min^–1^ at an elevated temperature of 140 °C, but an optimal reactor tube was necessary to secure the highest yield.

The esterification of benzoic acid under MW irradiation has been proven to be fast and efficient, and a few studies reported this reaction in the presence of acid catalysts.^30^ The most recent protocol of the continuous MW-assisted esterification of benzoic acid in the presence of PTSA was optimized at 140 °C on the basis of an MW-assisted protocol studied in a batch mode.^31^ On the basis of the order of reactivity of alcohols (*n*BuOH, *i*BuOH > *n*PrOH, *n*PentOH, *i*OctOH > EtOH > *i*PrOH), the authors accomplished the reaction in flow mode with a variable residence time of 20 to 30 min (Scheme 1B). The reaction was performed in a CEM Discover focused MW synthesizer equipped with a CEM 10 mL flow-cell accessory (irradiated volume 7 mL), wherein 35 mmol of benzoic acid was converted to its ester form in >95% yield. Another example of the esterification of levulinic acid using sulfuric acid or PTSA as the catalyst in MW batch mode and in conventional flow mode performed above the atmospheric boiling point of the solvent has been described.^32^ MW heating provided complete conversion at 120 °C in 5 min with catalyst loading of 2.5 mol % and a levulinic acid–ethanol ratio of 1:10 (Scheme 1C). To mimic the batch reaction conditions in conventional flow mode, two solutions of acid and of levulinic acid in ethanol were pumped in a 10 mL perfluoroalkoxy alkane (PFA) coil that could operate at up to 150 °C. When the reaction was performed with 2 mL/min flow, a conversion of 78% was observed; therefore, reducing the flow rate to 1 mL/min was found to be necessary to attain complete conversion in one pass.

**Scheme 2:**
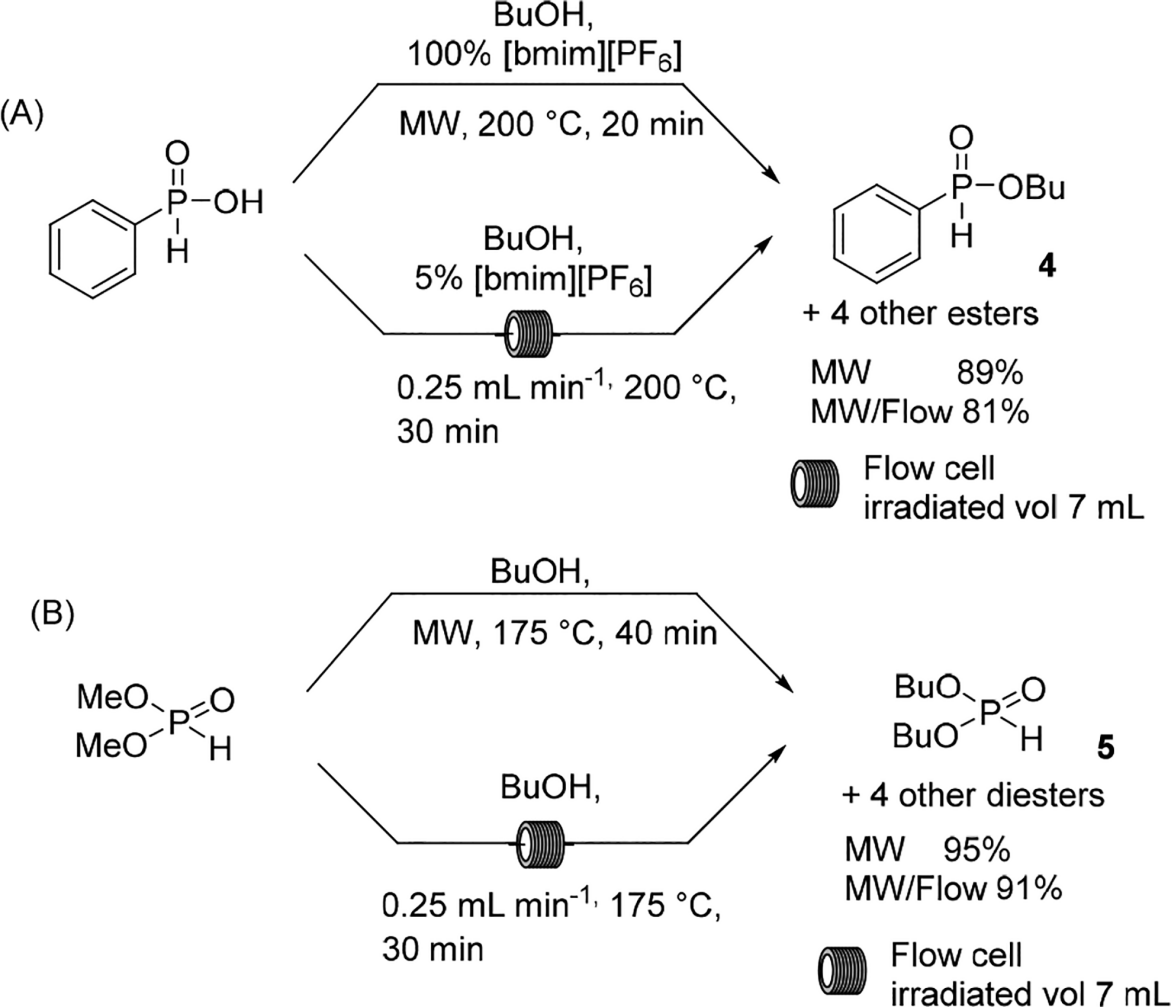
Organophosphorus Transformations

Large interest in organophosphorus transformations has garnered the attention of the MW-assisted protocol, and the esterification of phenyl-*H*-phosphinic acid took advantage of the high temperature, namely, in the alcoholysis of dimethyl *H*-phosphonate.^33−35^ Both MW reactions were performed and scaled up in MW/flow in a flow cell housed in a CEM MW reactor. The esterification was promoted by the addition of [bmim][PF_6_], which exhibited activity as an enhancer of MW irradiation, whereas transesterification generally did not require catalyst addition (Scheme 2A). Butanol reacted at 175–200 °C in 30 min in an MW flow cell of 10 mL, and the authors showed that roughly the same conversion was obtained in both the batch and flow modes (Scheme 2B).

**Scheme 3:**
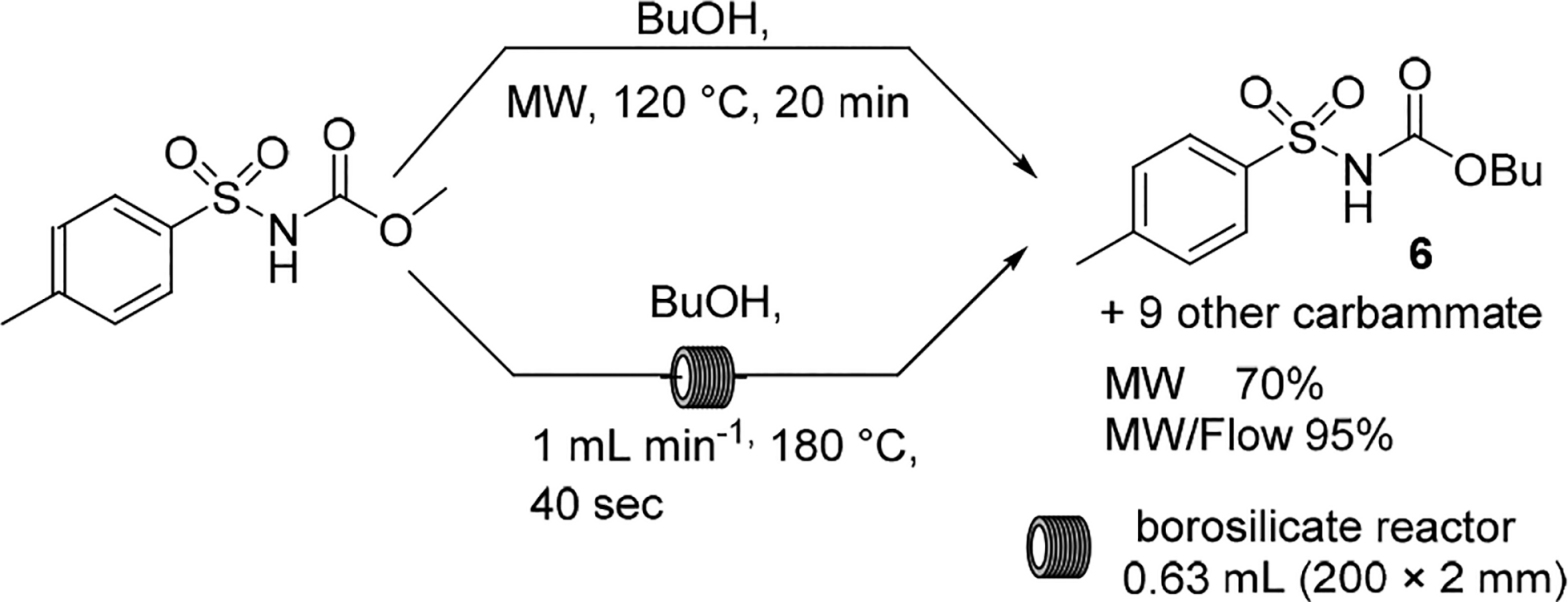
Transcarbamylation/Transesterification of Sulfonyl Carbamate

The investigation of the transcarbamylation/transesterification of sulfonyl carbamate evidenced that the MW flow procedures shortened the reaction time when compared to the MW procedure performed at the boiling point of the solvent. The eaction was shortened from 20 min to 40 s when butanol was heated from 120 °C in MW batch mode to 180 °C in MW flow mode (Scheme 3).^36,37^

**Scheme 4:**
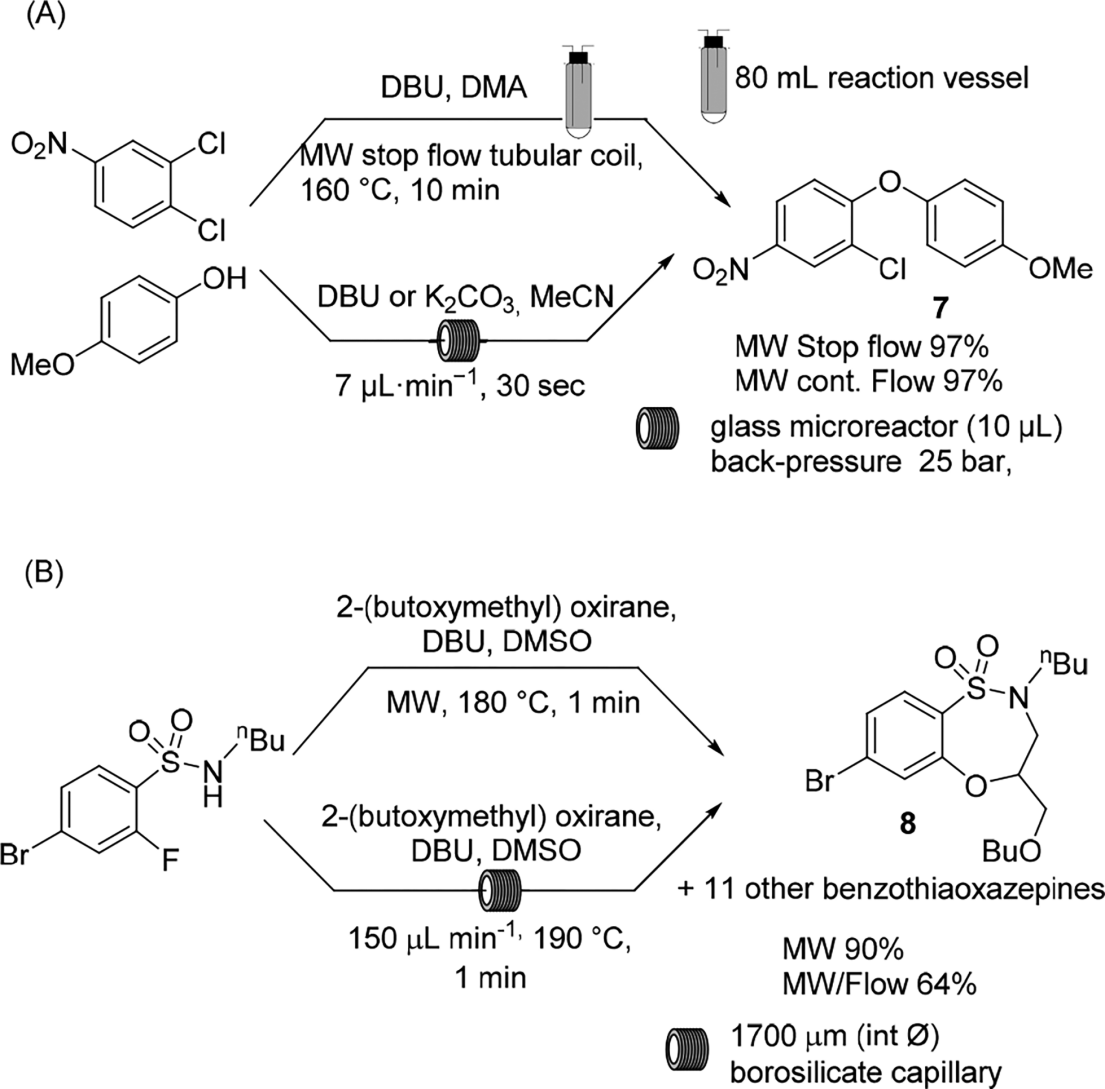
Scaling up of MW-Assisted SNAr

The SNAr reaction has been extensively studied by means of MW irradiation in a preliminary attempt to scale up the aromatic nucleophilic substitution of chloroarenes. The MW batch procedure could be adapted to a stop-flow continuous MW protocol.^38^ The reaction was performed between 1,2-dichloro 4-nitrobenzene and 4-methoxy phenol in an 80 mL reaction vessel (50 mL of operating volume) in the presence of an organic base (DBU) in DMA, and the vessel was filled and emptied along different lines (Scheme 4A, reaction conditions above). The automated repetition of the small-batch MW protocol ensured the large-scale production of product **7**. In 2011, the Watts group translated the methodology to a continuous-flow microreactor, enabling a reduction in the reaction time from 10 min to 60 s by means of a glass microreactor of 10 μL volume (Scheme 4A, reaction conditions below).^39^ Other advantages of the microreactor have been exploited because under pressure, a high-boiling solvent such as DMA could be replaced with MeCN. This could speed up the reaction outcome and the product isolation.

To extend the scope of MW continuous-flow SNAr, a two-step procedure via an epoxide opening/SNAr cyclization was reported by Organ et al. for the synthesis of benzofused sultams (Scheme 4B).^40^ The optimal protocol in batch mode was performed under MW irradiation in DMSO at 180 °C in 1 min, and DBU and *t*BuOK were selected as the optimal bases. Scaling up in continuous-flow mode by means of a single-mode Biotage Smith Creator synthesizer equipped with a 1700 μm (int Ø) borosilicate capillary allowed the production of 12 derivatives on a 2 to 3.5 g scale in a 55% average yield. To avoid clogging, the reactions were performed in the presence of DBU.

**Scheme 5:**
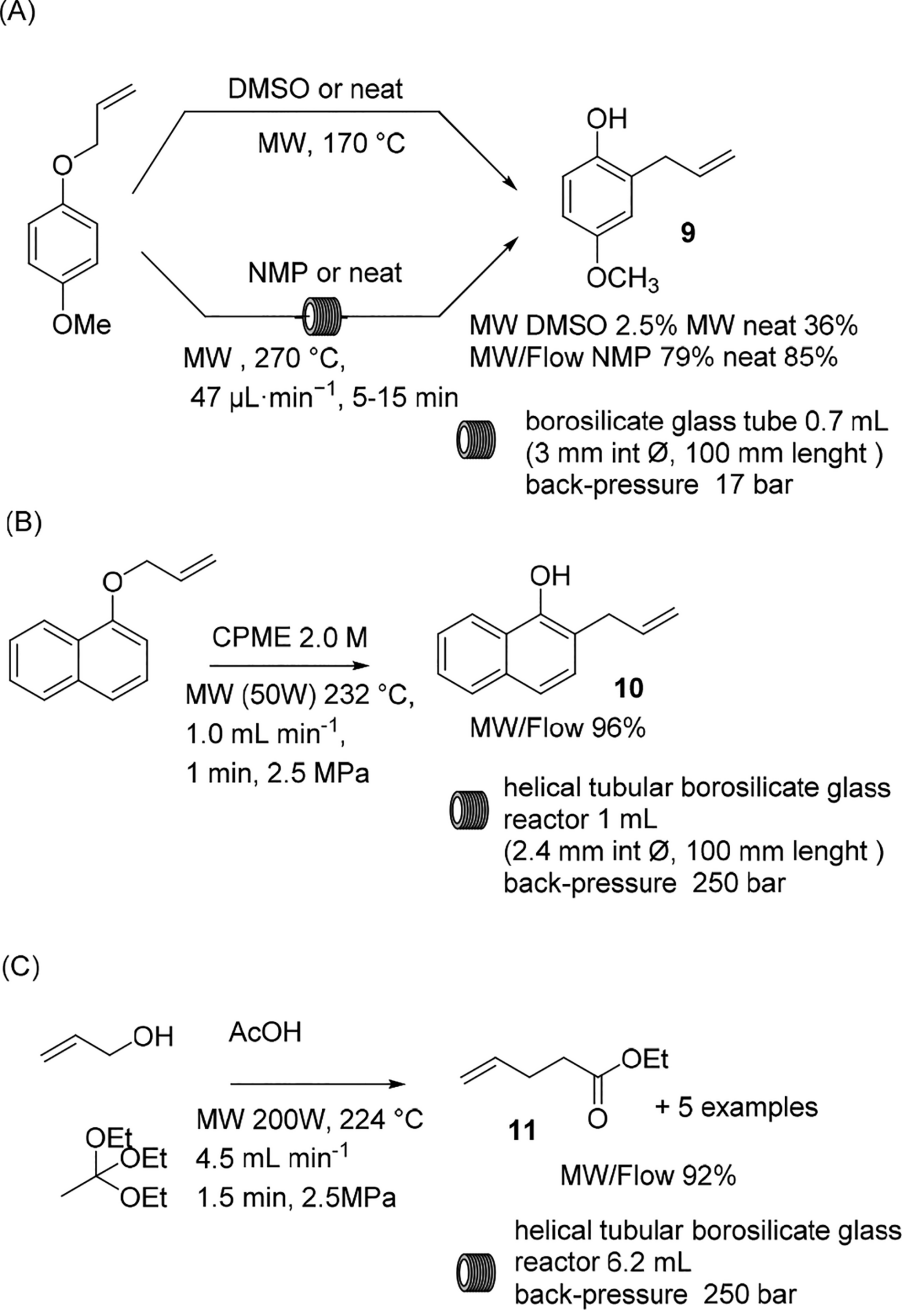
Claisen Rearrangement

Claisen rearrangement is accelerated under high-temperature and high-pressure conditions, and the efficacy of MW irradiation to enhance the reaction conversion has been exploited in continuous MW synthesis. Horikoshi et al. studied the influence of MW irradiation on the conversion of 1-allyloxy-4-methoxybenzene (Scheme 5A, reaction conditions above),^41^ as they observed that no significant improvement could be discerned when the reaction was performed under conventional conditions or under MW heating in DMSO. Neat conditions enabled the increase in the yield from 2.5 to 36%, and a two-fold yield improvement was observed compared to the reaction heated in an oil bath. A similar study was reported by Larhed et al. to access the applicability of a nonresonant MW system for the continuous MW/flow chemistry.^26^ As depicted in the Scheme 5A (reaction conditions below), the reaction has been performed in *N*-methyl-2-pyrrolidone (NMP) and neat at 270 °C, proving the efficacy of higher temperature conditions. In 5 to 15 min, the 2-allyl phenol was obtained in 78 to 85% yield, respectively, with a calculated throughput of 13.6 and 15.0 mmol/h.

Another study focused on a resonator-type MW reactor in which a helical tubular borosilicate glass reactor was placed within a rectangular resonant cavity where the electromagnetic wave intensity was maintained at the maximum. The Claisen reaction of allyloxy naphthalene was tested as a model reaction, and a detailed evaluation of the effects of the MW power, concentration, and flow rate were described by Koyama et al.^25^ An extremely efficient protocol was described in cyclopentyl methyl ether (CPME) with the substrate dissolved at an optimal concentration of 2.0 M in the presence of a back pressure of 2.5 MPa. The solution was heated to 232 °C and flowed at 1 mL/min with a residence time of 1 min to obtain the desired product in >95% yield (Scheme 5B). The scaling up of the reaction was validated with 11 g of starting material that reacted in 30 min, and a productivity of 20.26 g/h was attained.

The Johnson–Claisen rearrangement has been demonstrated to be a suitable option for the MW continuous-flow protocol. Allyl alcohol and triethyl orthoacetate can be converted to ethyl pent-4-enoate with an excellent productivity of 89.5 g/h (Scheme 5C). The desired product was collected in high purity with a residence time of ∼1.5 min. 10 to 25 mol % acetic acid was necessary to obtain the product in good yield while the reaction temperature was maintained at 224 °C.

**Scheme 6:**
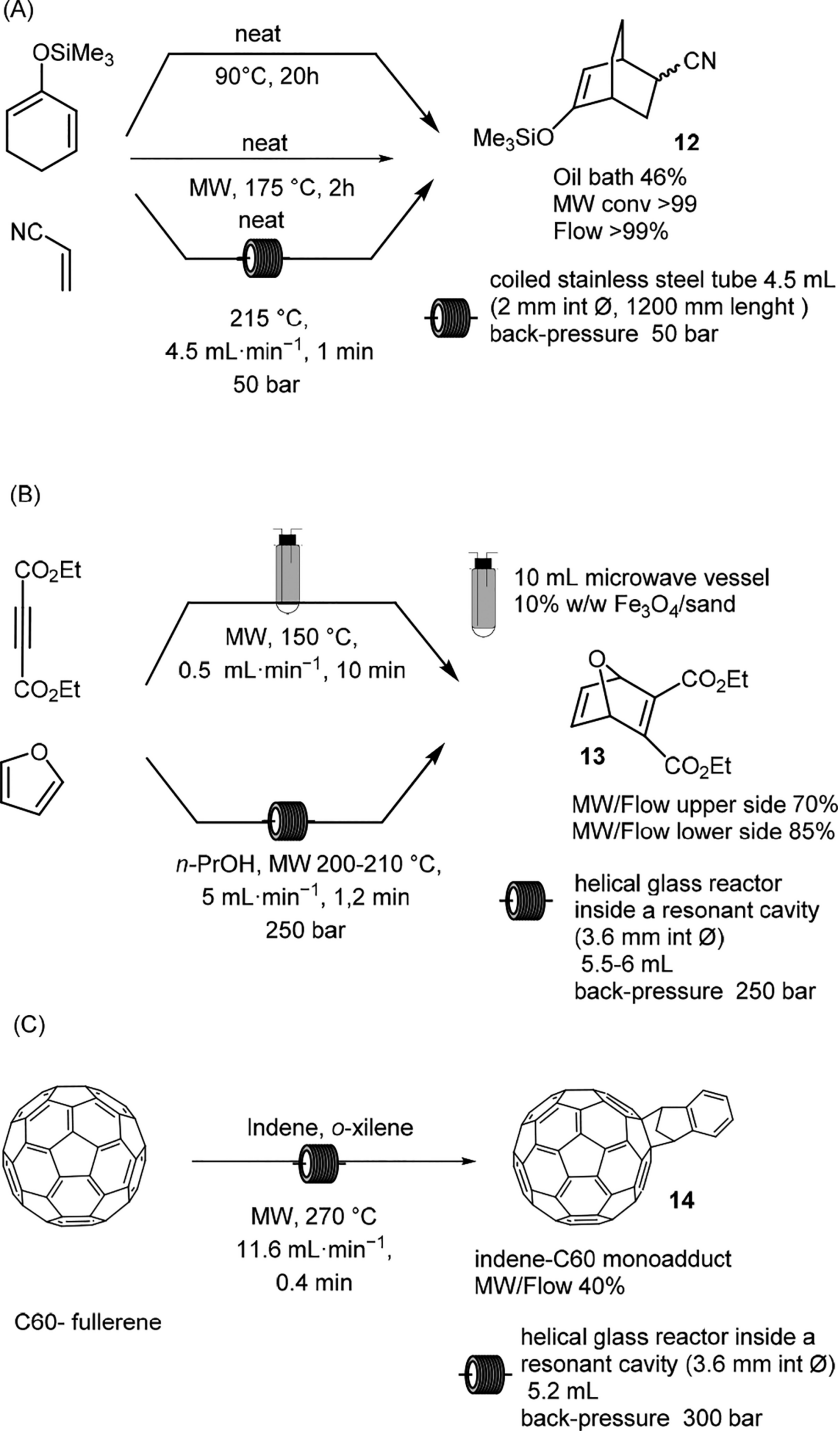
MW-Assisted Diels–Alder Reaction

Many examples of the Diels–Alder reaction have been reported in MW irradiation and in flow mode. The beneficial effect of MW irradiation was exploited to optimize the protocol and then was applied to the conventional flow mode. In 2012, Abele et al. reported the Diels–Alder reaction of (cyclohexa-1,5-dien-1-yloxy)trimethylsilane with acrylonitrile and observed that under MW irradiation the side reaction of acrylonitrile polymerization was highly reduced when compared to the conventional oil bath (Scheme 6A).^42^ Because the polymerization of acrylonitrile leads to microreactor clogging, the optimization of MW irradiation was performed at different temperatures up to 175 °C, and the authors observed that in 2 h, at 175 °C, acrylonitrile was completely consumed, but 6–8 mol/mol % of dienophile was still present. Therefore, they decided to translate the procedure in flow mode in the presence of excess flow. Working with a coiled stainless-steel tube of 4.5 mL, the reaction was scaled up in flow under solvent-free conditions. The reaction temperature was increased to 30 °C, and the reaction time was decreased to 2 min. When 2 equiv of dienophile was used, in 1.25 min, the reaction was complete, and no precipitation was observed. The conventional continuous flow was performed at 215 °C with a residence time of 60 s to generate 164 g of compound **12** in 45 min in 95% recovery.

The Diels–Alder cycloaddition between furan and diethyl acetylenedicarboxylate has been reported as a model reaction to assess the performance of two different continuous MW equipment setups. Two recent studies reported the reaction performed on meso scale. A 10 mL MW vessel tapered to a threaded inlet was used as a reactor in one case, whereas the other case exploited a helical tubular borosilicate glass reactor of 5.5 to 6.0 mL placed in the resonant cavity (Scheme 6B).^43,44^ In the first case, the reaction was performed at 150 °C for 10 min under neat conditions, which revealed that the MW/flow reaction yield was lower when compared with the conventional batch reaction. The 10 mL flow cell was therefore packed with a strong MW adsorber (10% w/w Fe_3_O_4_/sand) to increase the sample temperature, and an increase in the reaction yield from 60 to 70% was observed, but it was still lower than the reaction conventionally performed batch mode in the absence of the matrix (Scheme 6B, reaction conditions above).^43^ The second study reported the optimization of an MW-assisted flow Diels–Alder reaction in a resonant cavity. The optimal reaction conditions were obtained by varying the flow rate, the MW power, and the concentration of reagent.^44^ The highest yield of 85% was obtained with a short residence time (1.2 min) at the highest concentration of 4 M in *n*-PrOH at 200–210 °C measured as the exiting temperature (Scheme 6B, reaction conditions below). The authors observed that the advantages of continuous flow in this reaction were correlated to the rapid heating with a residence time shorter than 1.5 min as well as the rapid cooling to reduce the impact of the retro Diels–Alder reaction, which affects the reaction at an elevated temperature.

Furthermore, the Diels–Alder reaction has been successfully studied in the chemical derivatization of graphene and fullerene, and MW irradiation has been shown to improve its efficiency (Scheme 6C). A recent example includes the synthesis of an indene-C60 monoadduct and an indene-C60 bis-adduct. Because of their high open cell voltages, these products are used in polymer solar cells, and the scaling up of their synthesis is highly desirable. To achieve maximum productivity, the authors optimized the reaction at a temperature of up to 270 °C with the highest concentration of indene in *o*-xylene. The flow rate was maintained at 11.6 mL/min (residence time of 0.4 min), and the productivity was found to be 1.07 g/h.^45^

**Scheme 7:**
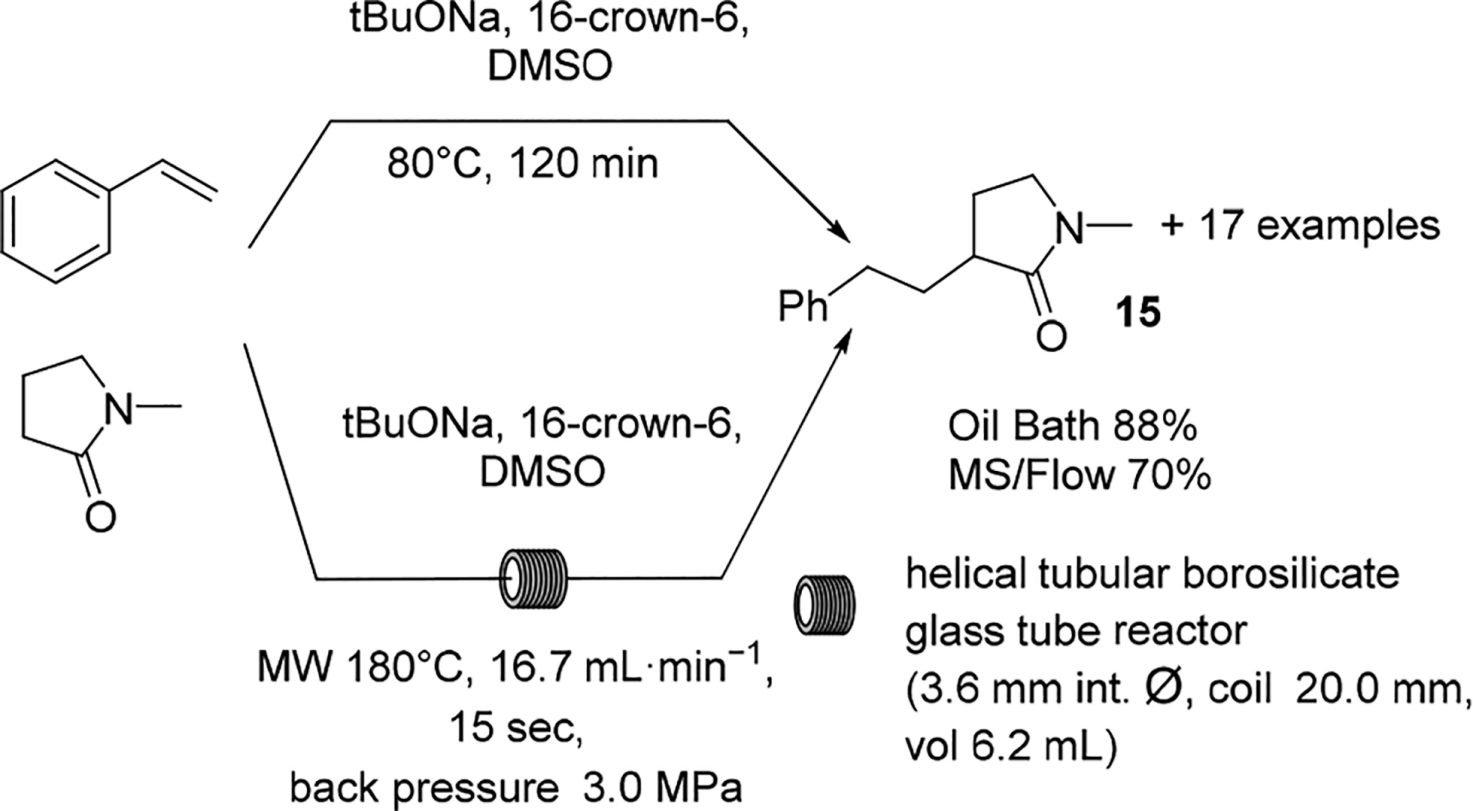
C-Alkylation of *N*-Alkylamides

With the aim to secure arylbutanamides on a large scale, a pharmaceutically relevant substructure, the C-alkylation of dimethylacetamide with styrene has been studied using MW irradiation and scaled up in MW continuous flow mode (Scheme 7).^46^ Interestingly, an optimized protocol allowed the use of *t*-BuOK instead of a stronger base such as BuLi with superb results in the synthesis of a large array of derivatives. When the reaction was scaled up in flow mode in a resonant MW cavity, excellent results were obtained, and the reaction could be scaled up to reach a productivity of almost 65 g/h. A high selectivity of mono- versus dialkylated product was obtained (10:1 mono/dialkylated).

## Applied Homogeneous and Heterogenoeus Catalysis

MW-assisted synthesis has been largely applied to organometallic reactions,^47−50^ including a review in 2019 on the continuous-flow Suzuki–Miyaura and Mizoroki–Heck reactions describing the efficacy of MW heating in scaling up of the reaction protocols in continuous mode.^51^ In addition to palladium-catalyzed synthesis, a few examples of homogeneous organometallic catalysis with other metals have been scaled up under MW continuous flow mode, and several examples have documented the attempts to optimize the scalable processes in MW batch synthesis.

Ru-catalyzed metathesis has been reported under MW irradiation,^52,53^ and an example of the scalable procedure was proposed by Grela et al. with the aim to contribute to the scaling up of the synthesis of Apremilast, an orally active drug for the treatment of psoriatic arthritis and psoriasis; nevertheless, the reaction has been reported on only a small scale.^54^

**Scheme 8:**
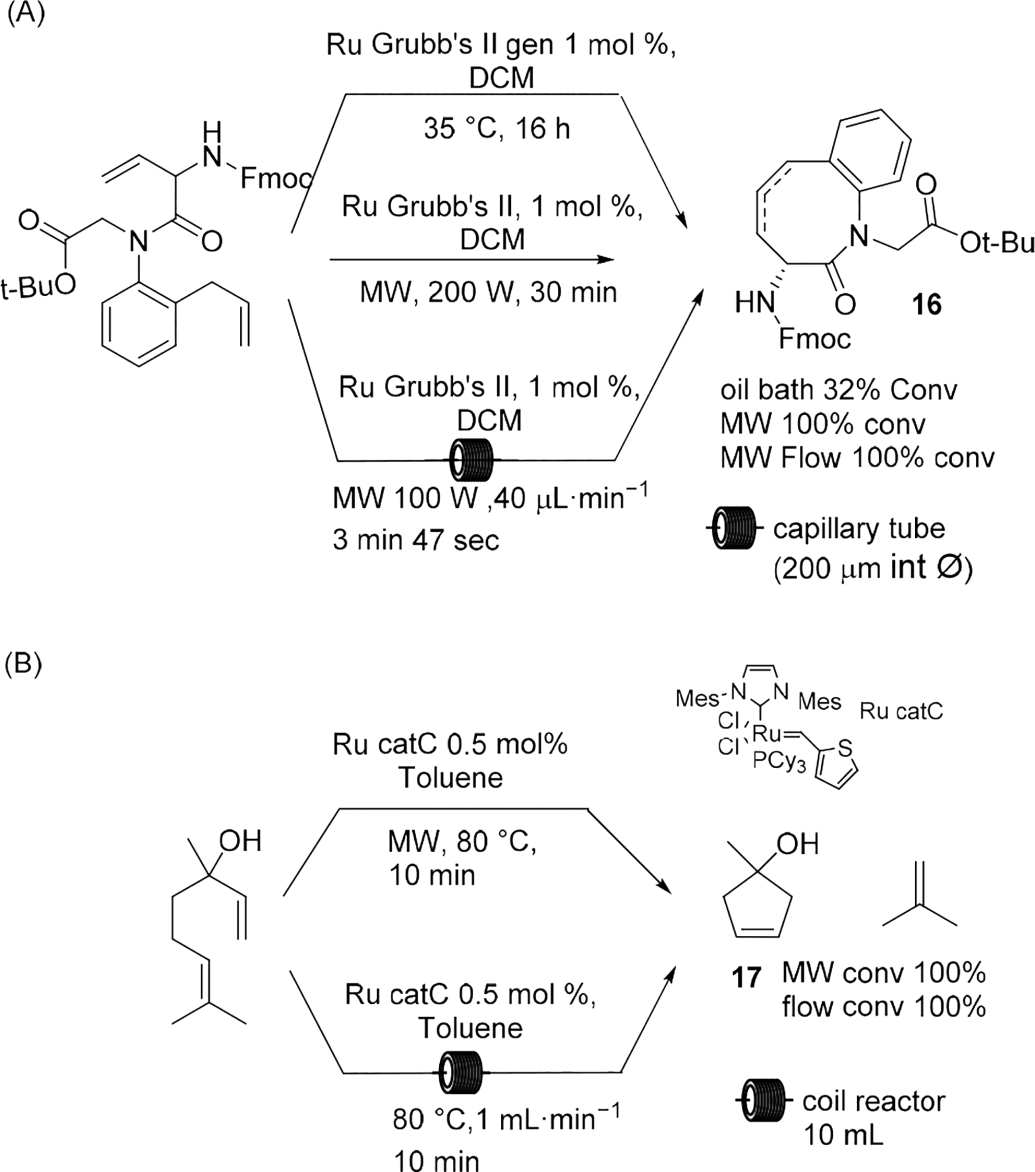
Ruthenium-Catalyzed Ring-Closing Metathesis

An example of the nonconventional scaling up of ring-closing metathesis (RCM) was proposed by Organ et al.^55^ The generation-II Grubbs catalyst was tested, and the authors compared the conventional conditions, MW batch irradiation, and continuous flow irradiation with a capillary-based flow system in the synthesis of compound **16**. As depicted in Scheme 8A, interestingly, the reaction was performed in DCM, an MW transparent solvent (tan δ 0.040); nevertheless, the study showed that MW irradiation showed higher conversion when compared with the conventional conditions, suggesting a direct effect on the reagent and catalyst. As already demonstrated, the expectations based on the temperature can be exceeded when MW irradiation is exploited with polar substrates in nonadsorbing solvents.^56^ MW energy can thus be considered as an important variable for selectivity attainment in organic synthesis.

Recently, another example of ruthenium-catalyzed metathesis for the ring-closing of linalool and of citronellene was studied by Leadbeater et al. (Scheme 8B).^57^ The reaction was performed in MW batch irradiation and then scaled up to mesoscale conventional flow irradiation in the presence of a modified second-generation Grubbs Ru catalyst. The successful reaction ensued in the presence of 0.5 mol % of catalyst at 80 °C in 10 min for the ring closing of linalool, whereas 20 min was necessary for citronellene. The results obtained in the MW batch irradiation could be perfectly transferred to conventional flow irradiation.

**Scheme 9:**
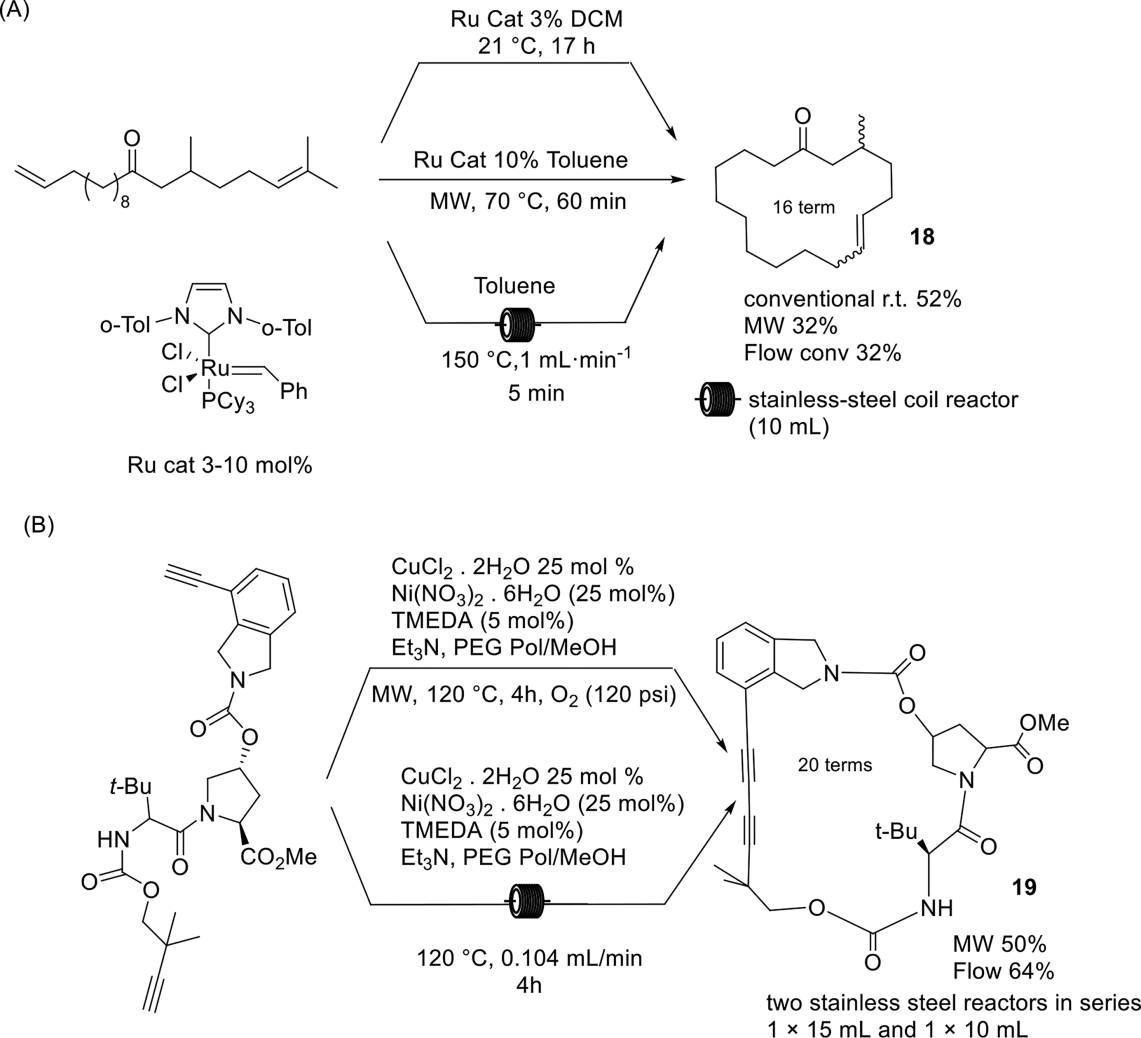
Macrocyclization

An unsuccessful study on the applicability of MW batch and flow irradiation in macrocycle (C-16) RCM revealed that the higher temperature is responsible for the decomposition of the Ru catalyst and that flow techniques trapped the ethylene produced during the reduction, thus generating a number of side reactions (Scheme 9A).^58^ Similar results were obtained in the synthesis of a C-16 macrocycle having a strong musky odor at 70 °C in toluene under MW irradiation and flowing catalyst and reagent at 1 mL/min (residence time of 1 min). When the reaction was performed conventionally in DCM at room temperature, the reaction rate was quite slow, but higher yields were obtained.

Another example focused on the synthesis of a macrocycle under MW irradiation and under flow conditions was described by Collins in 2017.^59^ The proposed new approach aimed to synthesize Vaneprir, a protease inhibitor for hepatitis C treatment (Scheme 9B). The study is based on a catalytic macrocyclization by means of a phase separation strategy wherein poly(ethylene)glycol (PEG) lipophilic aggregates are formed, and the slow diffusion of the precursor toward the polar methanol solvent preserves the reaction outcome, avoiding the side reactions. The macrocyle was obtained by a copper-catalyzed Glaser–Hay oxidative coupling in the presence of a nickel cocatalyst, and the optimization of the protocol under MW irradiation allowed shortening of the reaction time for scale up in conventional flow mode. As depicted in Scheme 9B, the reaction yield under nonconventional conditions increased from 50 to 64% with a reaction time of 4 h; when compared with the conventional conditions, the reaction was shortened by 20 h while maintaining a comparable yield.

The application of continuous MW protocols in the preparation of metallic nanoparticles of small regular-size nanocatalysts has been the object of large interest, as summarized in recent reviews.^8,60−62^ The beneficial effect of MW irradiation on solid-supported catalysts is well-recognized,^63−66^ as is the impact of flow chemistry on process intensification.^9^

Because of their large industrial interest, innovative protocols in catalytic oxidation or reduction reactions represent one of the most appealing exploitations of MW chemistry in batch mode and flow mode.^67,68^

MW-assisted hydrogenation reactions cannot be easily performed on a large scale because of the limitations due to gaseous hydrogen. By means of a specially designed MW oven that allows a single reaction chamber to perform single or multiple reactions at temperatures up to 300 °C and pressures up to 199 bar with a gas inlet that enables the operation in a modified atmosphere (Synthwave Milestone), hydrogenation reactions can be performed safely under MW irradiation and H_2_ pressure (1 bar). A recent example of Au-catalyzed levulinic acid hydrogenation was performed at 50 bar of H_2_ at 200 °C to produce 1,4-pentandiol in high yield with high selectivity.^69^ Large batch hydrogenation under MW irradiation has not been reported in the literature, however.

**Scheme 10:**
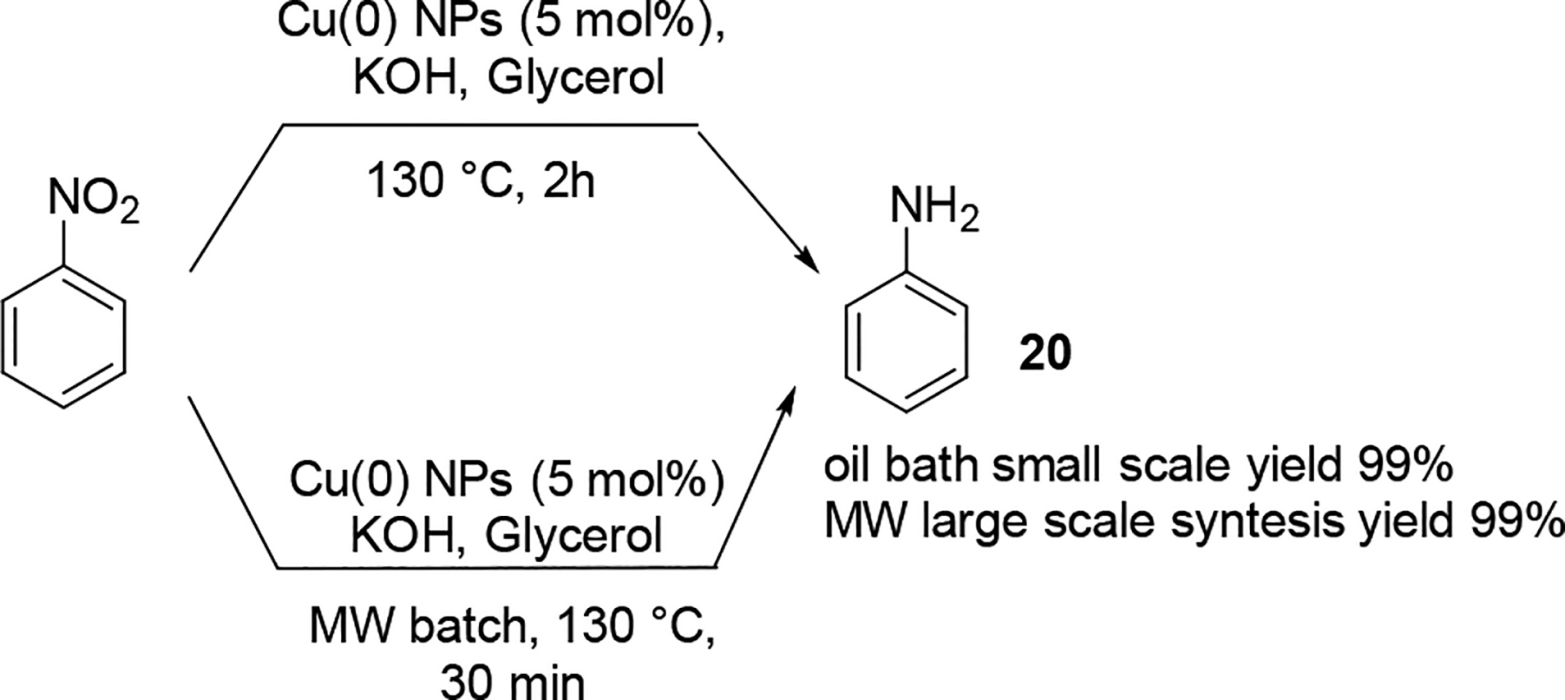
Large-Scale Cu(0) Nanoparticle-Catalyzed Reduction of Nitrobenzene in Conventional and in MW Batch Mode

**Figure 5:**
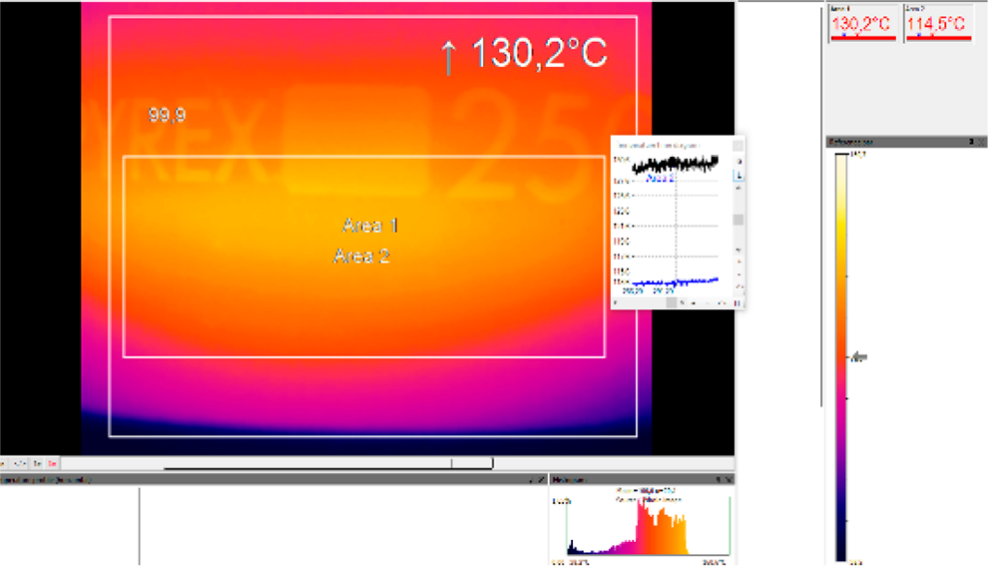
Density of temperature recorded by an IR camera in an MW-irradiated reduction of nitrobenzene Cu(0) nanoparticles cat. Reproduced with permission from ref (70). Copyright 2020 The Authors.

Reduction by transfer hydrogenation has addressed this limitation, and recently, an example of the copper-catalyzed (Cu(0) nanoparticles) reduction of nitrobenzene in glycerol was described in MW large batch mode (Scheme 10).^70^ Because glycerol is a biobased and eco-friendly solvent with a high MW adsorption capacity and a low vapor pressure, and because the reaction demanded a high temperature, a large-scale synthesis of the MW batch mode was studied. The reaction was performed on an almost 1 L scale in a pilot MW reactor in 95% yield, and the study demonstrated that the sample temperature in the reaction mixture was between 130 and 115 °C, with a high absorbance of delivered MW power when the power was maintained constant (Figure 5).

**Figure 6:**
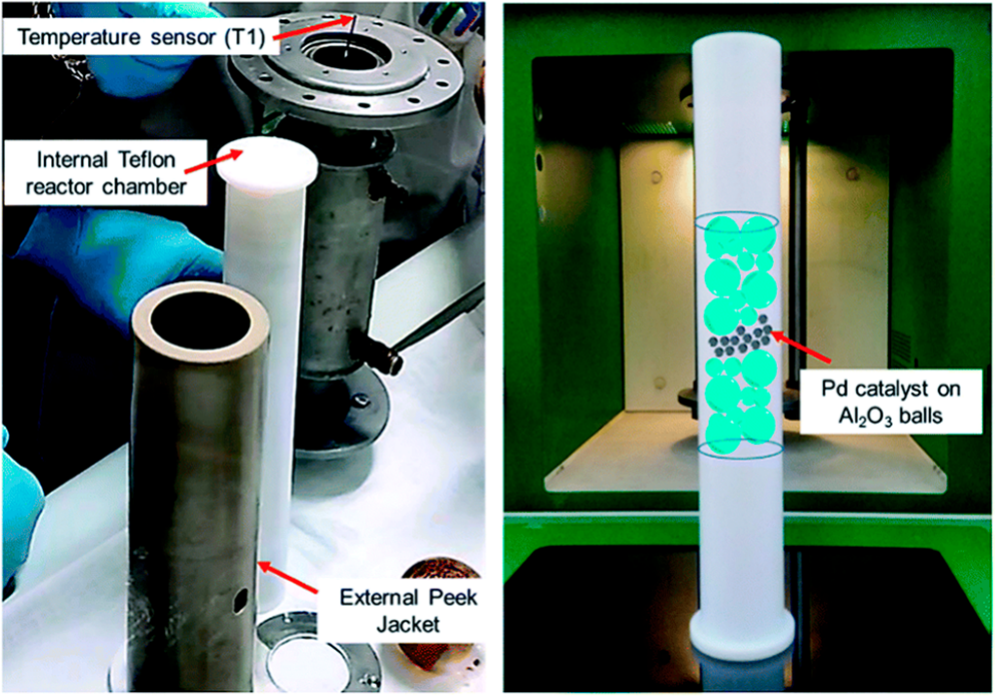
Pd/Al_2_O_3_-catalyzed hydrogenation of butynol in flow: MW reaction chamber.

**Scheme 11:**
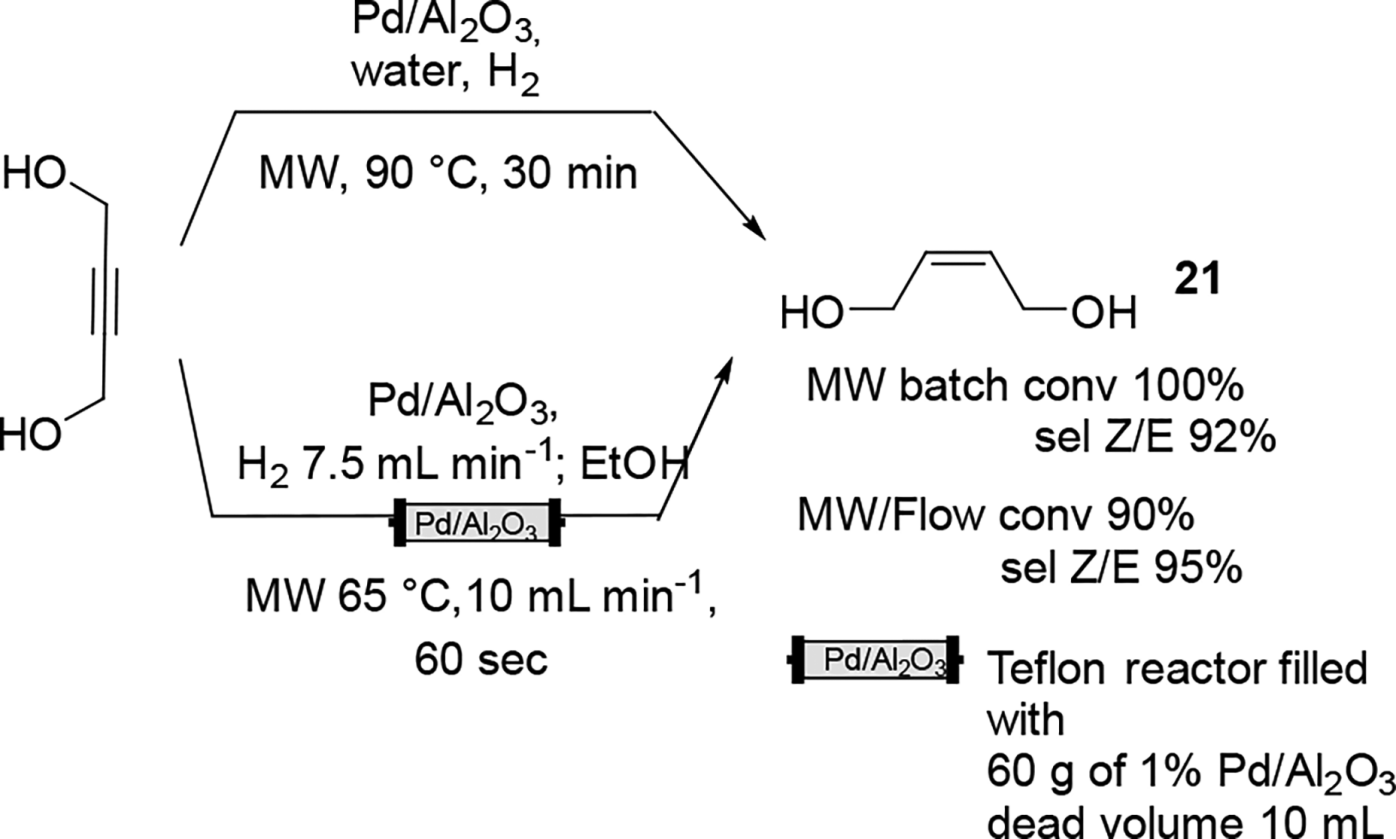
Semyhydrogenation of 1,4-Butynediol in MW Batch Mode and Continuous Mode

An original setup of the MW-assisted semihydrogenation of alkyne in continuous-flow (gas/liquid) mode is shown in Figure 6.^71^ The reaction of the reduction of butyndiol was performed in MW batch mode, wherein a continuous and a controlled flow of 7.5 mL of H_2_ was maintained to achieve not only high conversion but also high selectivity toward the *cis*-2-buten diol (Scheme 11). The selected catalyst was based on the solid supported Pd nanoparticle on an alumina sphere, which displayed good stability, and gradual deactivation was observed after 20 L of flowing reaction mixture. The productivity of the scaled-up procedure reached 70 mol g M^–1^ h^–1^.

**Scheme 12:**
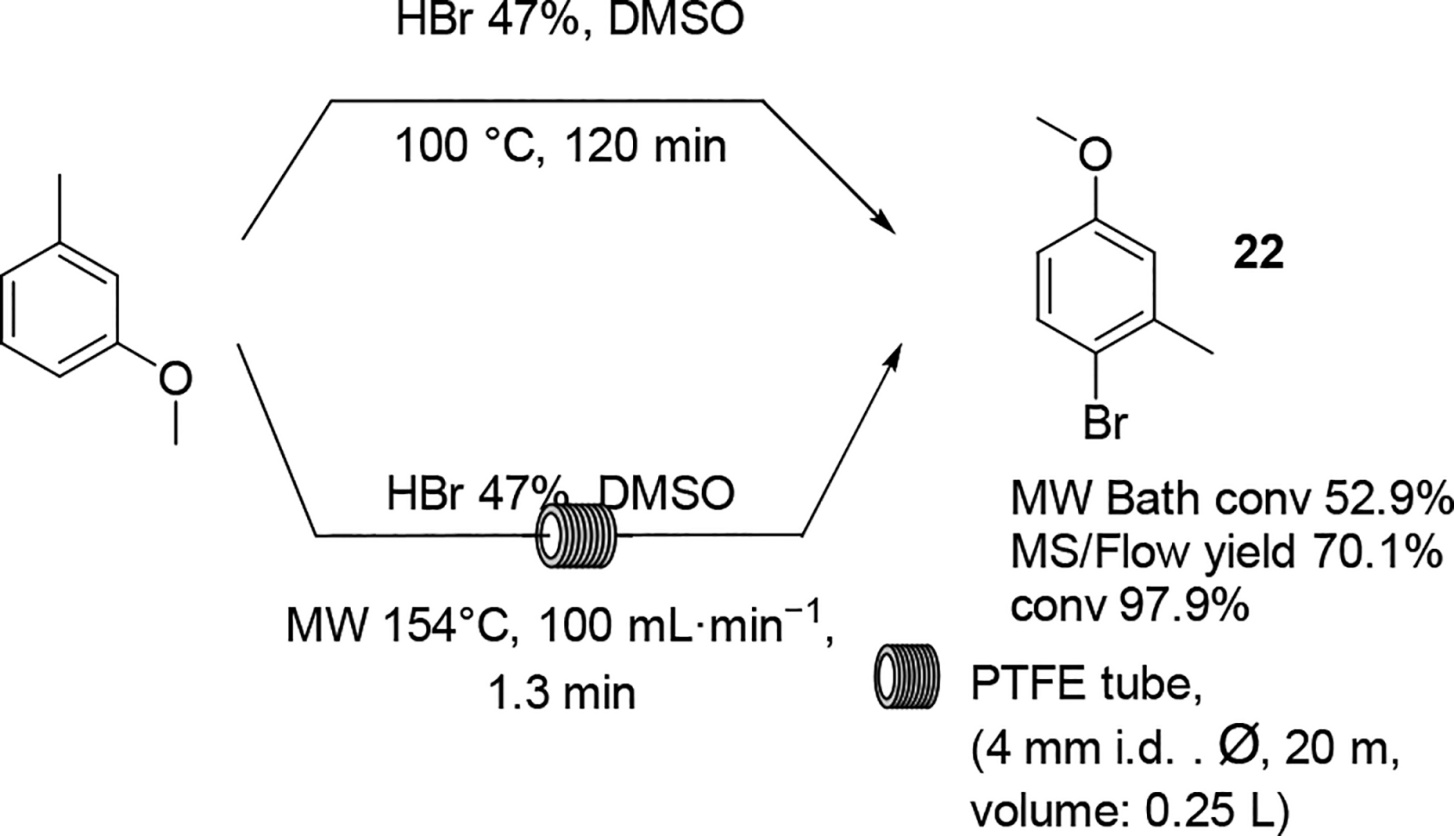
Oxidative Bromination of 3-Methyl Anisole in MW Batch Mode and Flow Mode

The synthesis of aryl bromides via oxidative bromination with DMSO and hydrogen bromide is an efficient and inexpensive reaction that can be performed on a kilo scale.^72^ An attempt to perform the scaling up by MW irradiation in batch mode and in continuous flow mode illustrated the beneficial influence of high temperature (Scheme 12).^73^ When the reaction was performed in flow mode, the desired 4-bromo-3-methylanisole **22** could be collected in a short retention time of 1.3 min, and the synthesis was performed on an almost half a kilo scale. In 5 h, 0.32 kg of product could be obtained in 79% yield.

**Scheme 13:**
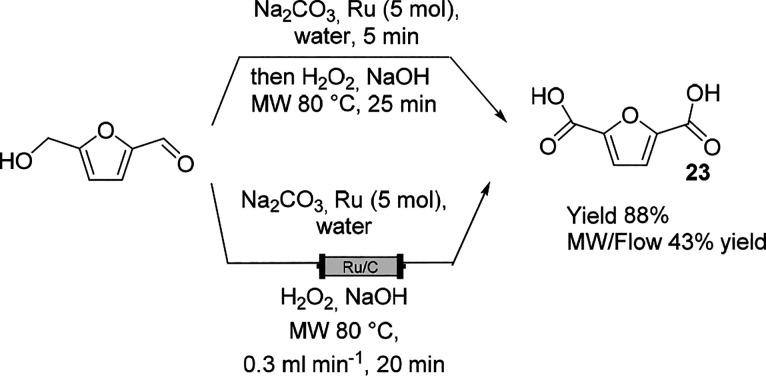
Ru-Catalyzed Oxidation of HMF

The use of MW irradiation in the conversion of biobased products has gained interest in the last several years.^74^ MW irradiation has been successfully applied in glycosylation reactions,^75^ and a continuous two-step process was studied in 2011 for the preparation of adenosine by glycosylation and deprotection.^76^ Several appliances under process intensification conditions have been reported as nonconventional protocols for the catalytic oxidation of 5-hydroxy furfural (HMF) produced from cellulose.^77^ Recently, aqueous H_2_O_2_ and air were deployed as oxidants in a commercial Ru/C catalyzed oxidation of HMF (Scheme 13) by means of different nonconventional technologies such as MW irradiation and continuous-flow MW irradiation.^78^ The reaction displayed excellent results in MW batch mode under optimized conditions, but a lower yield in continuous flow mode was obtained due to the instability of the catalyst. Not only the leaching of Ru but also the disruption of the porous structure of the catalyst was discerned by the performed analysis after usage.

## Synthesis of Heterocycles and Multicomponent Reaction

A green and sustainable approach to the synthesis of heterocycles is well documented.^79,80^ Because of the broad interest in medicinal chemistry and in polymer synthesis and material science, the synthesis of heterocycles has been explored, taking into account not only the use of technologies to save time and energy but also the deployment of greener solvents and even catalyst-free protocols.

**Scheme 14:**
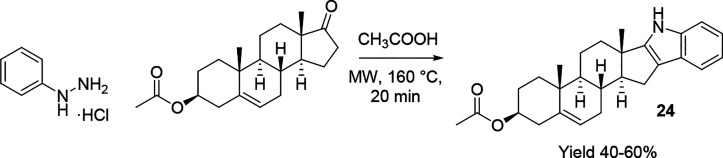
MW-Assisted Fisher Indole Synthesis for the Undergraduate Laboratory

The synthesis of indole has been intensely investigated because of its importance in tryptamine-based pharmaceuticals. In addition, diverse methods for its preparation via Fischer synthesis have immensely benefitted from the use of MW irradiation. Synthetic protocols for indole synthesis were proposed in MW batch mode with a homogeneous^81^ or heterogeneous acidic catalyst^82^ and scaled up in MW continuous mode.^83^ Because of its high efficiency and purity, MW-assisted Fischer synthesis has been practiced in the undergraduate laboratory starting from dehydroepiandrosterone acetate and phenyl hydrazine hydrochloride salt in glacial acetic acid (Scheme 14).^84^

**Scheme 15:**
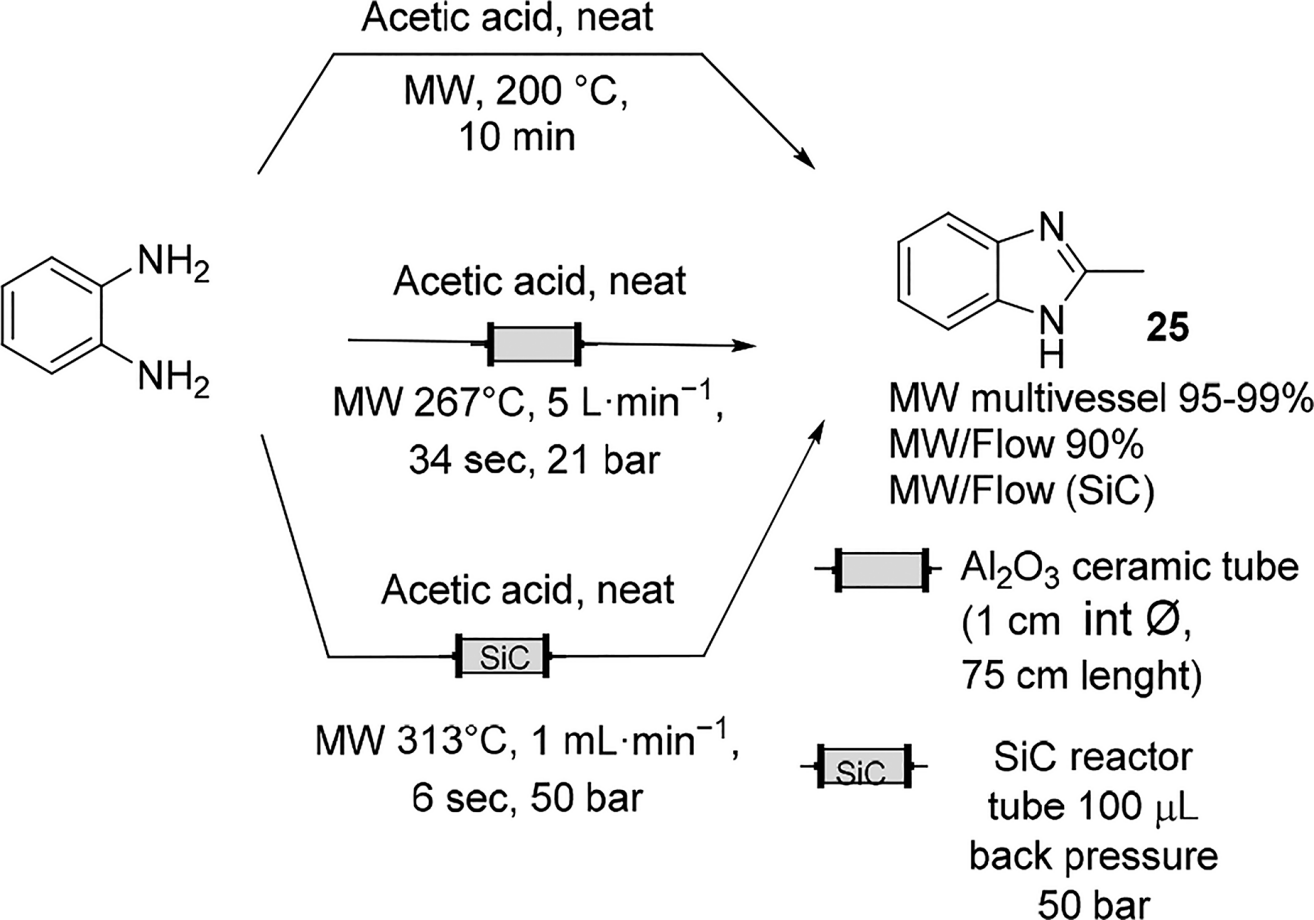
Synthesis of Imidazoles

The synthesis of methyl benzimidazole under MW irradiation from *o-*phenylenediamine in acetic acid is a simple condensation procedure that has been studied by different research groups (Scheme 15). Because a higher temperature favors attaining complete conversion in a shorter time and because acetic acid has a high loss tangent of 0.174, MW irradiation could accomplish the reduction of reaction time. Scaling up of this reaction under solvent-free conditions with a moderate to high scale of production has been documented. In 10 min, the synthesis was performed in parallel in a multimode MW instrument with multivessel rotor systems and a total reaction volume up to 960 mL (16 Teflon vessels of 60 mL each) by Kappe et al.^85^ A few years later, the same group in collaboration with Clariant Produkte Deutschland reported the kilo scale production of methyl benzymidazole in continuous mode by means of an MW system operating at high temperature/high pressure processing 20 L/h.^22^ In 2014, Organ et al. presented another example of this reaction performed in a continuous-flow MW reactor in which the back-pressure regulators guaranteed no pressure fluctuations during the reaction and the system was equipped with a SiC reactor tube to shorten the reaction time to 6 s at 313 °C.^86,87^ In all of the cases operating with a high-temperature/high-pressure process, the yield was maintained at >90%.

**Scheme 16:**
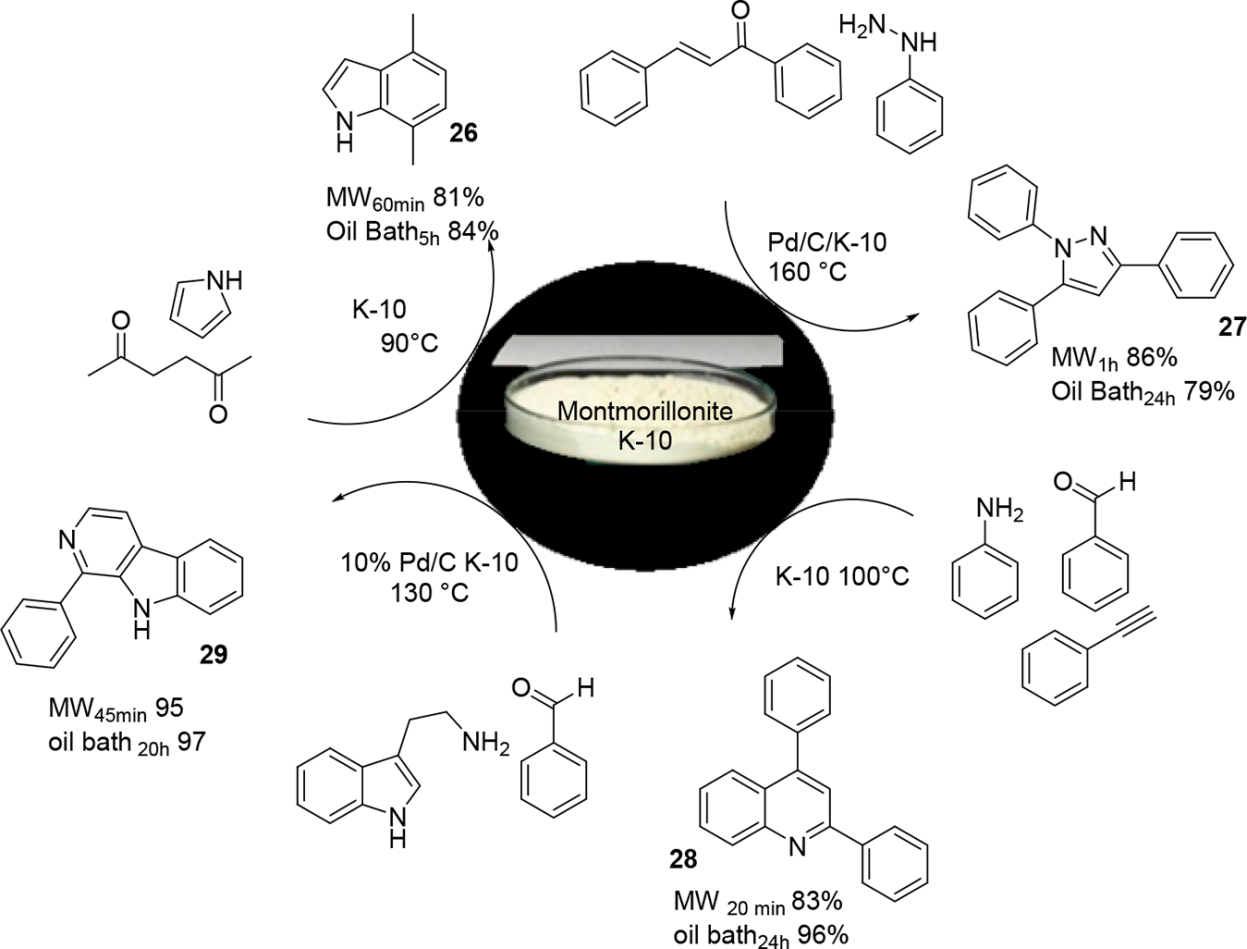
Montmorillonite-Catalyzed Multicomponent Reactions

The beneficial effect of MW irradiation on the heterogeneously catalyzed synthesis of heterocycles has been of great interest in view of the large applicability of the heterocycle in pharmaceutical chemistry. MW irradiation facilitates a reduction in reaction time, and by deploying multicomponent reactions, high atom efficiency can be reached. Additionally, a number of successful protocols have appeared in the literature, and it is not always that the reaction yield by MW irradiation is higher than that under conventional conditions. As a proof of this concept, in 2014, Török et al. studied six different reactions catalyzed by semisynthetic montmorillonite K-10, a good MW absorber, and measured the energy consumption (kWh/% yield). The electrophilic annulation of pyrrole, the synthesis of substituted pyrazole by the domino condensation–cyclization–aromatization reaction, the domino Pictet–Spengler cyclization–dehydrogenation reactions, and the multicomponent domino cyclization–aromatization reactions were compared (Scheme 16).^88^ Pyrazole synthesis from chalcone (product **27**) and the synthesis of quinoline **28** and of carbazole **29** reached a high *E*_oil bath_/*E*_MW_ ratio, with a value of 5.6, 9.1, and 5.7, respectively. When the indole was obtained by electrophilic condensation, the reaction represented an exception, and the conventional heating revealed a lower energy consumption with respect to MW irradiation. The quinoline synthesis was scaled up by 40 times to 17 g; interestingly, the MW irradiation produced the final product in 90% yield, whereas the conventional heating afforded a 70% yield, and the *E*_oil bath_/*E*_MW_ ratio was 7.6, underlining the higher energy efficiency of the MW-assisted reaction. On the basis of the reported results, the conventional heating generally consumed more energy when compared with MW irradiation in heterogeneously catalyzed heterocycle synthesis; nevertheless, the reaction should be studied on a case-by-case basis.

The use of MW irradiation in multicomponent organic synthesis has been well exploited.^89^ Because of their potential to limit the number of synthetic steps while increasing the molecular complexity and diversity, multicomponent reactions have been largely used in pharmaceutical chemistry.

The Ugi reaction has been introduced for the preparation of multifunctional peptides and heterocyclic compounds, as summarized in a recent review.^90^ MW heating contributes to the efficacy of the Ugi reaction because of the high polarity of the intermediates. Furthermore, the MW irradiation power as well as the solvent selection have been demonstrated to influence product selectivity.^91^ Because of the long reaction time, MW irradiation also shows good advantages in increasing the reaction rate.

**Scheme 17:**
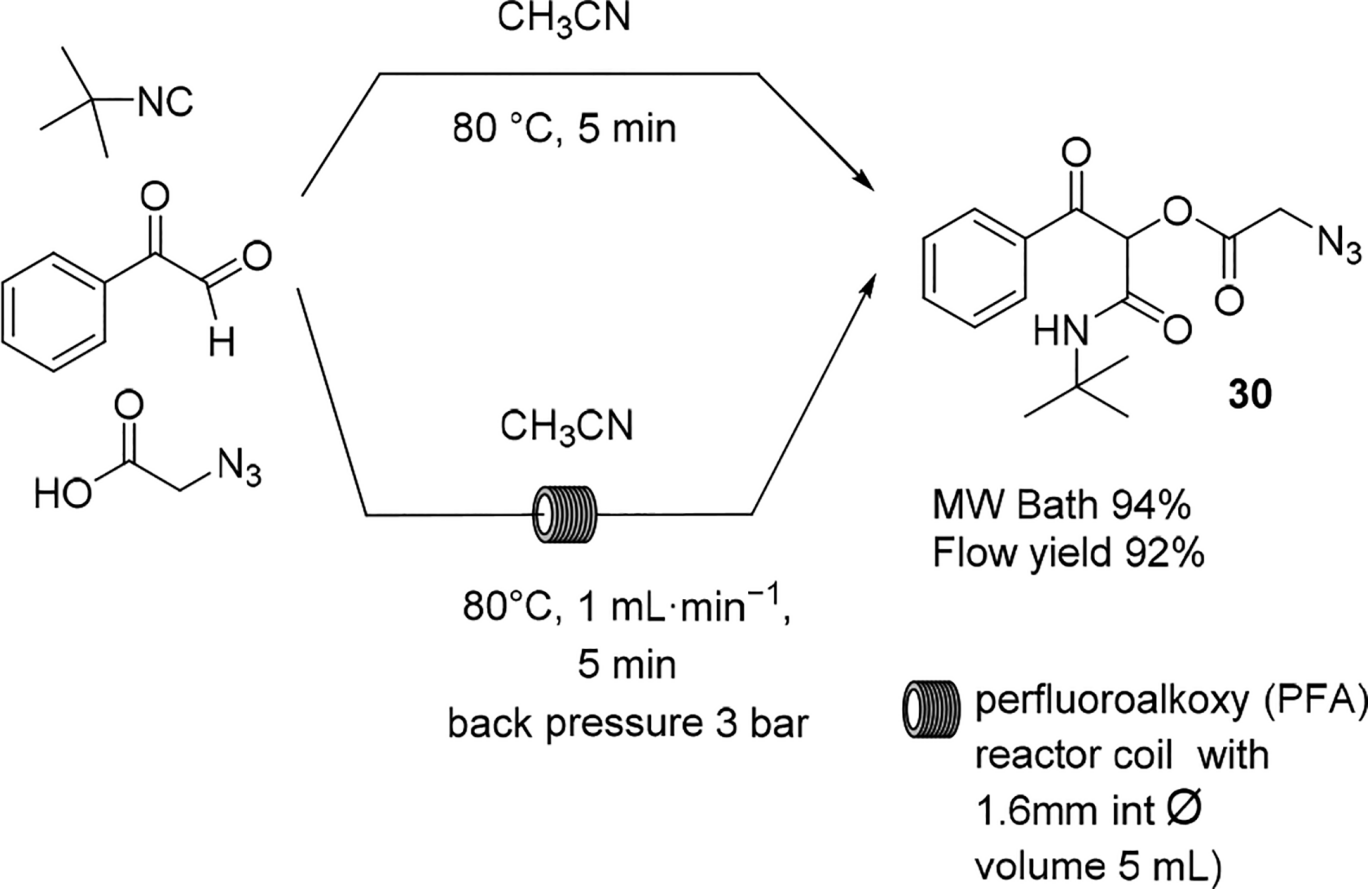
Isocyanide-based Multicomponent Passerini Reaction

In 2015, Salvador et al. reported an MW-promoted sequential Ugi reaction/Cu-catalyzed alkyne azide cyclization to produce cyclo peptoids that was scaled up in conventional flow mode.^92^ In 2019, the same group described another scalable procedure for the multicomponent isocyanide-based Passerini reaction optimized in the MW system (Scheme 17).^93^ From the optimization studies in batch mode, it was observed that in toluene and CHCl_3_, the conversion was very low or near-zero because of their low MW dielectric heating properties. In acetonitrile, the reaction efficiently delivered the desired product. When performed in the conventional flow setup, the α-acyloxy ketone derivative was obtained in comparable yield with a productivity of 0.312 g/min.

Because of the well-known pharmacological activity of dihydropyridines for the treatment of thrombosis, atherogenesis, and cardiovascular disease, the four-component Hantzsch synthesis has been extensively studied under conventional and unconventional conditions.^94,95^

**Scheme 18:**
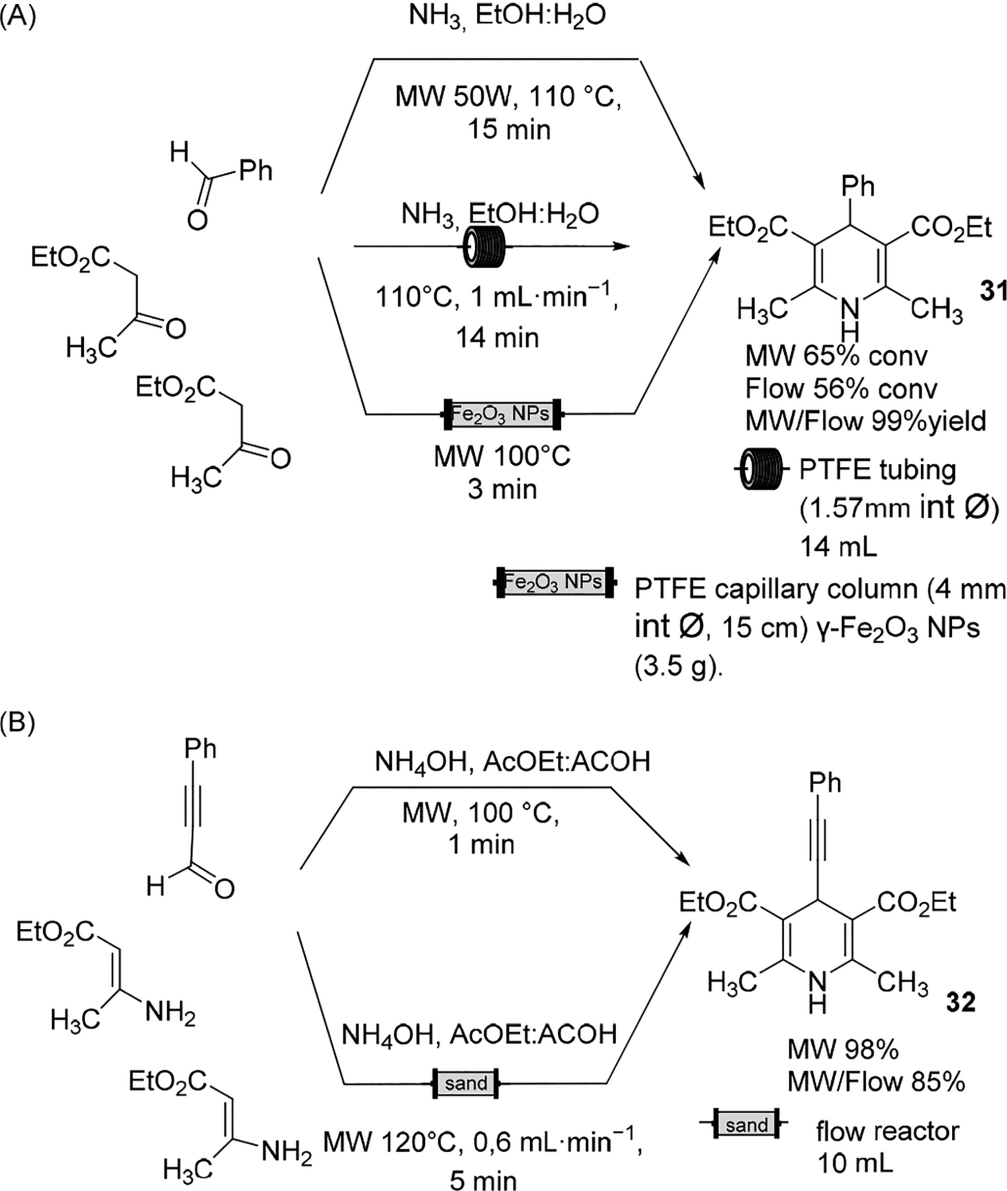
Hantzsch Multicomponent Synthesis

The first example of scaling up in the continuous MW irradiation of 4-aryl-1,4-dihydropyridines was reported in 2001, deploying a domestic MW oven in which a round glass reactor of 65 mL was placed along the circumference of a glass plate, and the synthesis was performed in an aqueous hydrotrope solution (50% sodium *p*-toluene sulfonate) to maintain a homogeneous reaction medium.^96^ More recently, the limits of Hantzsch multicomponent synthesis in continuous mode were underscored in two publications. In the first study, the reaction was performed with MW irradiation on a 100 mmol scale of the three components, and the reaction conversion was compared with that of the conventional flow mode (Scheme 18A).^97^ In addition to the fact that the conversion was lower in the flow mode when compared to the MW batch mode, the low solubility of the of 4-phenyl-1,4-dihydropyridines in ethanol imposed the authors to add ethyl acetate to the reaction mixture once out from the heated aluminum block. The energy consumption in Hantzsch synthesis was measured, and comparing 100 mmol reaction scale heated for 15 min, the energy efficiency of the MW batch mode measured in Wh was more than two times that in the flow mode.

Another study confirmed the same trend by performing the reaction under MW irradiation at 140 °C for 10 min and in conventional flow mode.^98^ Formaldehyde was used instead of benzaldehyde, and an attempt to reduce the molar ratio was also pursued. The yield in conventional flow mode was lower than that in MW batch mode, ranging from 82% in MW batch mode to 68% in flow mode. Better results were observed when the synthesis of enamine in situ was avoided and acetoacetate was replaced with ethyl β-aminocrotonate and phenylpropargyl aldehyde instead of formaldehyde (Scheme 18B). When the reactions in batch mode and in continuous MW irradiation mode were compared, higher yields were attained, and the flow synthesis reached a higher yield. The better performance of the multicomponent Hantzsch reaction in continuous mode was achieved in the presence of a heterogeneous catalyst (γ-Fe_2_O_3_ nanoparticles) packed in a polytetrafluoroethylene (PTFE) capillary column.^99^ Because the magnetic nanoparticles catalyzed the multicomponent reaction,^100^ a 98.7% yield was obtained at 100 °C in 3 min residence time, but surprisingly, a large excess of catalyst was necessary to obtain full conversion (Scheme 18A).

**Scheme 19:**
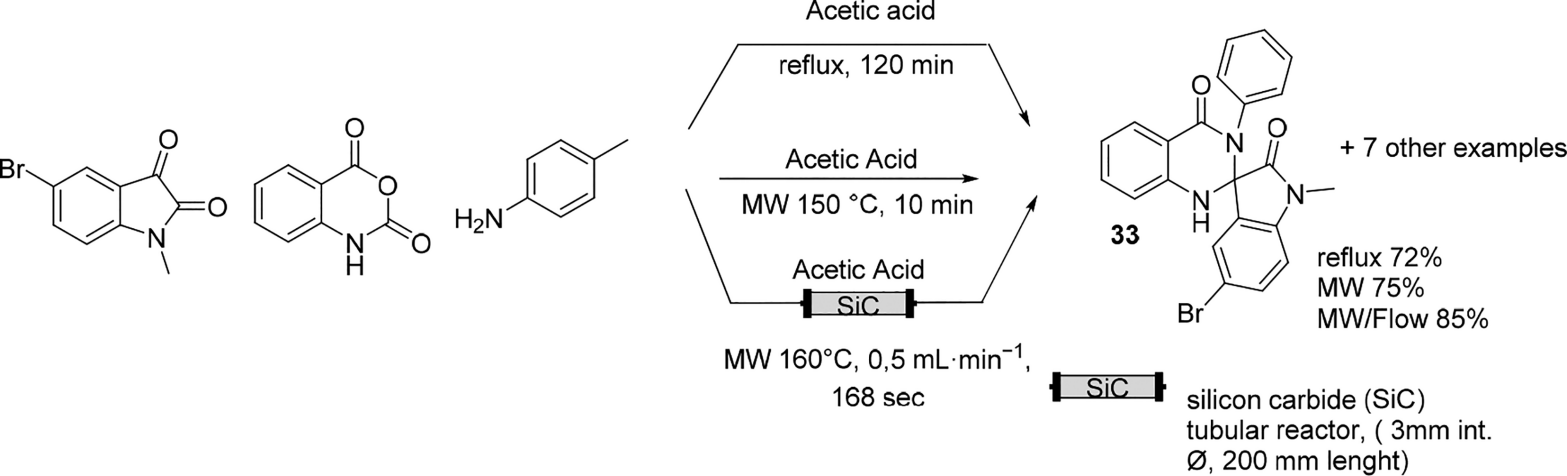
Three-Component Spiro-Oxindole Synthesis

An interesting example of three-component spiro-oxindole synthesis was proposed by Larhed et al.^101^ As depicted in Scheme 19 the influence of MW irradiation in combination with continuous flow in a silicon carbide tubular reactor shortened the reaction time and increased the yield.^87^

## Stereoselectivity in Microwave-Assisted Synthesis

High-temperature and high-pressure chemical transformations may suffer from low stereochemical control; nevertheless, nowadays, several studies have reported the stereoselective synthesis under MW irradiation conditions. Because selectivity is a crucial objective in organic synthesis, a careful evaluation of the reaction conditions is always warranted, and in addition to solvent selection, an appropriate study of temperature and time allows kinetic versus thermodynamic control. The possibility of controlling the regio/stereoselectivity by choosing the appropriate conventional MW heating is an attractive field of research.

**Scheme 20:**
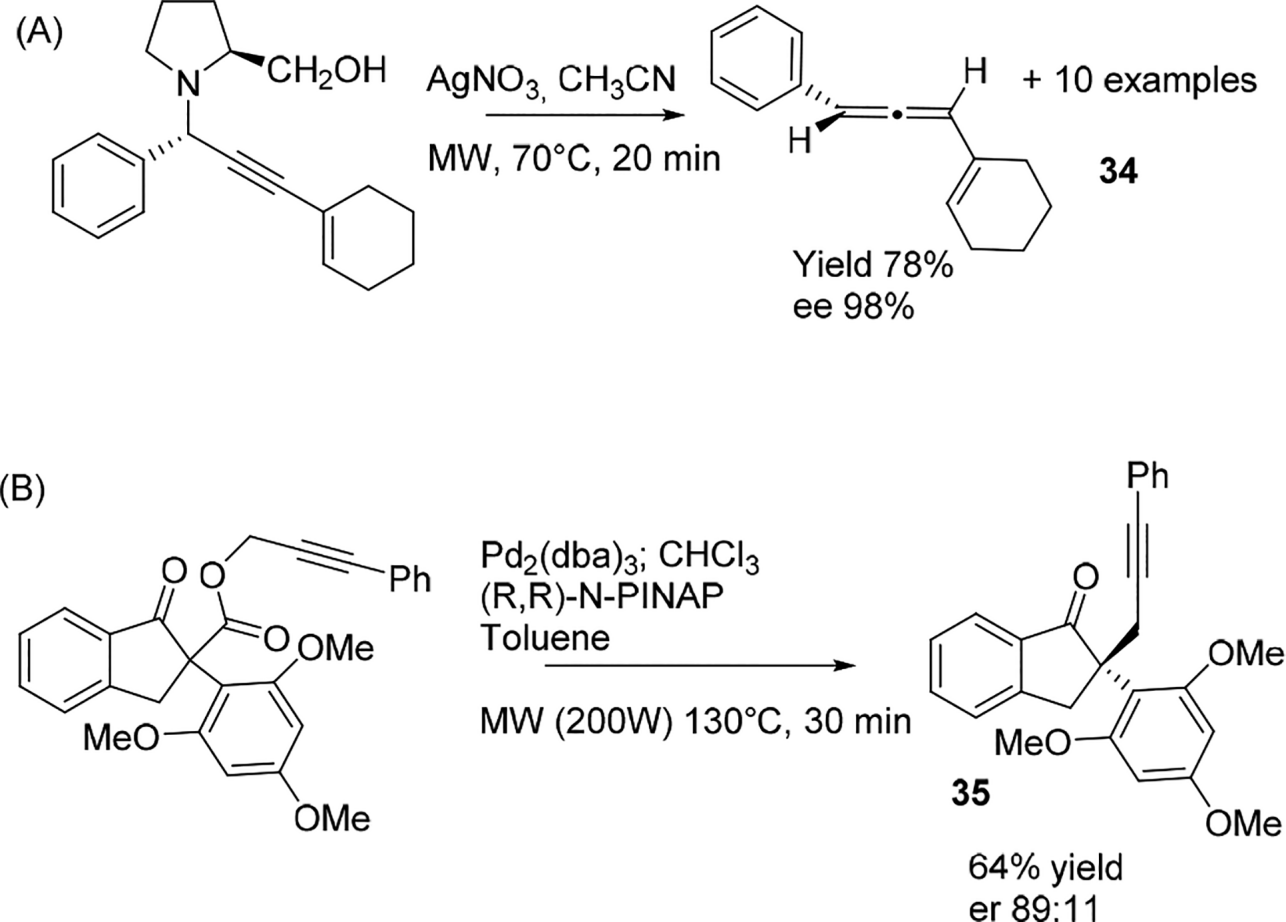
Stereoselective Synthesis

As depicted in Scheme 20A, vinylallenes can be obtained enantioselectively from the alkyne in the presence of a chiral auxiliary under MW irradiation.^102^ Optically active propargylammine, in which (*S*)-prolinol was the stereogenic group, reacted in the presence of AgNO_3_ in acetonitrile at 70 °C under MW irradiation (50 W) in 20 min. The reaction proceeded to generate the desired allene derivatives in 70% average yield, exemplified by a series of 11 cases with high stereoselectivity (79 → 99% ee).

Another recent example reported the palladium-catalyzed decarboxylation/alkylation of propargyl cyclopentane carboxylate (Scheme 20B) to produce the final product **35** in 64% yield with 89:11 er in presence of (R,R)-N-PINAB.

**Scheme 21:**
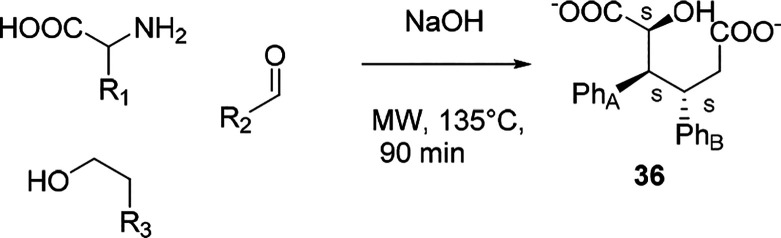
Multicomponent Diasteroselective Synthesis

**Scheme 22:**
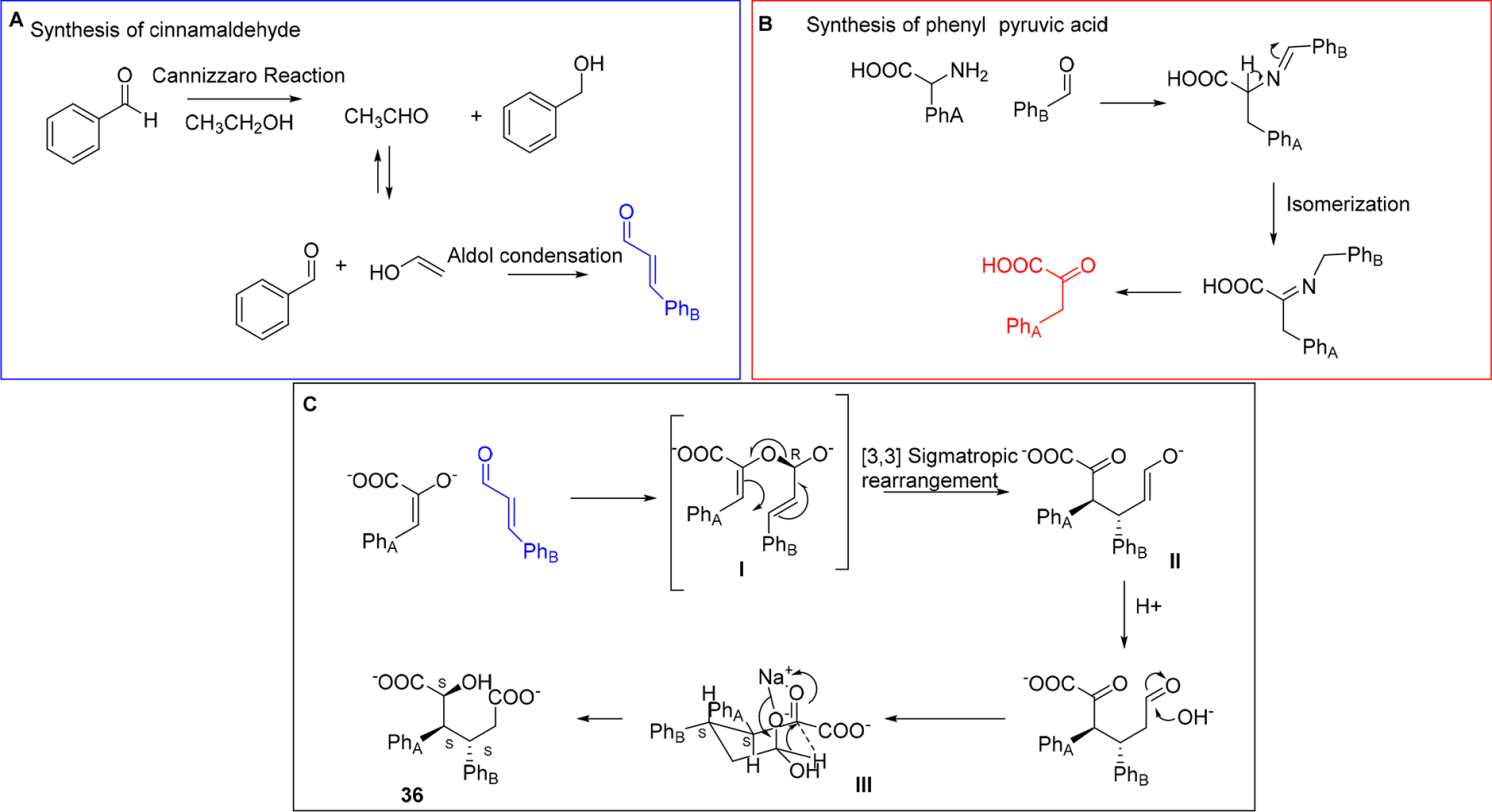
Mechanism of Multicomponent the Diasteroselective Synthesis of Dicarboxylic Acid **36**

Unforeseeable results were recently obtained by Repo et al. when the condensation of l-phenylalanine and benzaldehyde was performed in alkaline ethanol under MW irradiation (Scheme 21).^103^ A single crystalline compound with the structure of a dicarboxylic acid **36** was isolated and characterized by X-ray crystallography as a racemic mixture of RRR and SSS enantiomers. Intriguingly, both the pyruvic acid derivative and cinnamaldehyde were obtained by means of MW heating wherein the first one was formed by the condensation of an amino acid with benzaldehyde followed by isomerization and hydrolysis, whereas the second one could be synthesized via a Cannizzaro-type reaction followed by aldolic condensation (Scheme 22). The diastereospecificity of this reaction has been driven on the basis of a [3,3]-sigmatropic rearrangement that involves cinnamaldehyde and phenylpyruvic acid. From the hydrated derivative of aldehyde **II**, it is assumed that the Na^+^ ion favors a chairlike conformation intermediate that delivers the final product by hydride transfer.

**Scheme 23:**
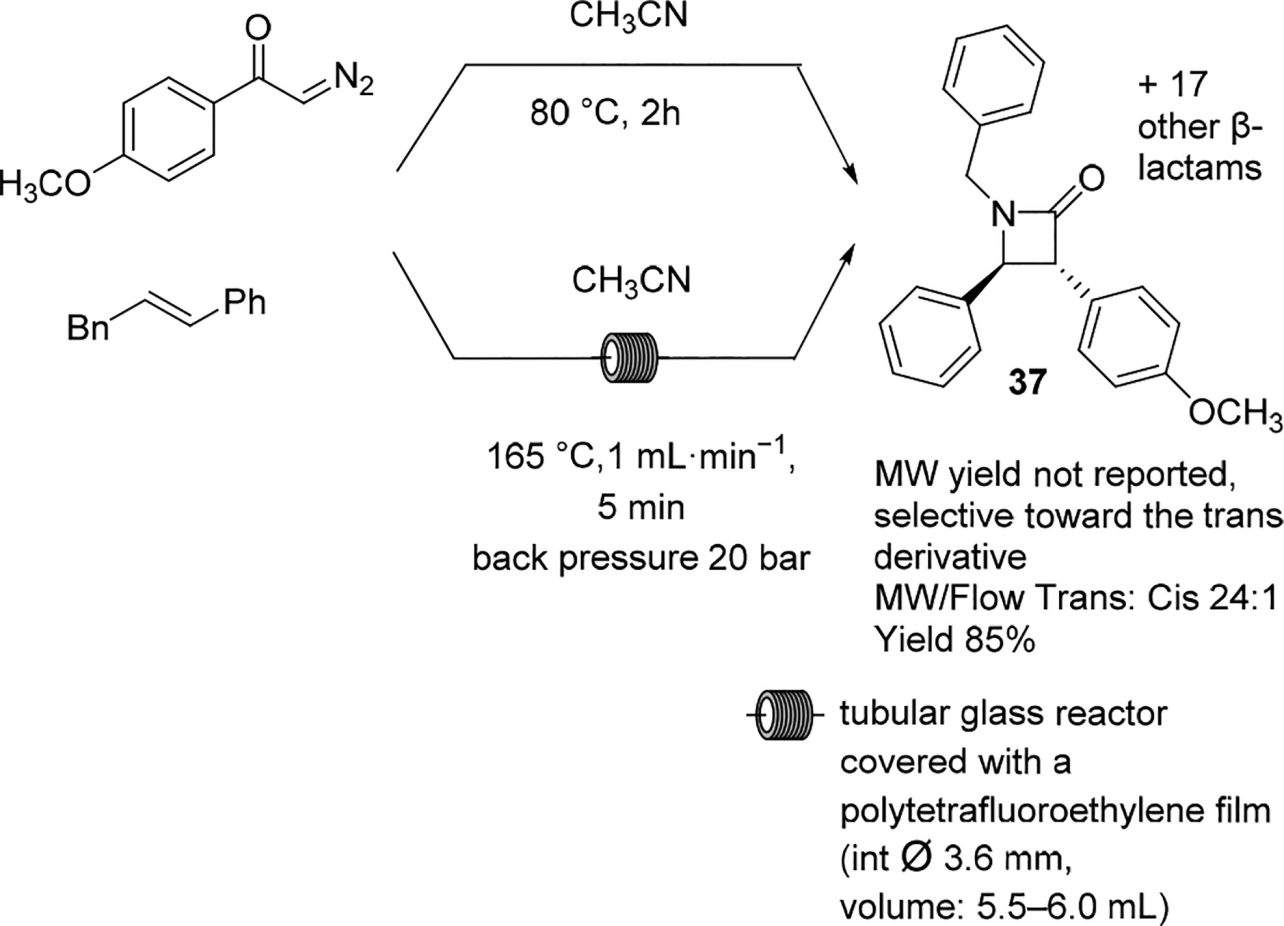
Wolff–Staudinger Reaction of β-Lactams

The Wolff–Staudinger reaction affords the formation of β-lactams via the [2 + 2] cycloaddition of ketenes with imines. The diastereoselectivity for the trans-substituted reaction has been studied in MW batch mode^104^ as well as under continuous MW operation.^105^ Because the protocol is considered problematic due to the release of gaseous nitrogen and in view of the high temperature, continuous MW mode is an appealing alternative to the batch synthesis. A set of 18 different compounds with yields ranging from 33 to 85% were synthesized with general selectivity toward the trans configuration. Electron-poor imines gave the lowest yield and stereoselectivity, whereas electron-rich imines were efficiently converted to the trans isomer. In general, high temperature and MW heating provided the optimal diastereoselectivity in the Staudinger reaction (Scheme 23). Aimed to predict the stereoselectivity or to rationalize it, density functional theory (DFT) calculations were also included in the study.

## Conclusions

On the basis of the recent literature, numerous advances in MW technologies have been reported. The possible applications of MAOS are extremely broad. When highly adsorbing polar solvent, reagents, or catalysts are deployed, a tremendous reaction rate increase is observable under MW irradiation conditions. Nevertheless, protocols may be affected by a number of variables related to the diverse equipment and different technologies used for the measurement of temperature. The multitude of reactions require a case-by-case study, as an automatic green label for MAOS is not warranted. However, MACOS represents the best setup option for any scaling up efforts geared toward the potential industrialization of dielectric heating. This also requires a careful measurement of the dielectric properties, namely, the permittivity, of reagents/reactants, solvents, and catalysts. The new age of MAOS is far from the empirical approach of the first enthusiastic practitioners and may address the strict rules of good manufacturing practice (GMP) production.
